# Activatable fluorescent ratiometric probes for early diagnosis and prognostic assessment of acute kidney injury

**DOI:** 10.1126/sciadv.aea1654

**Published:** 2025-10-22

**Authors:** Ni Li, Zeyang Liu, Chunda Chen, Hongjing Jiang, Yiting Liu, Dalong Ni

**Affiliations:** Department of Orthopaedics, Shanghai Key Laboratory for Prevention and Treatment of Bone and Joint Diseases, Shanghai Institute of Traumatology and Orthopaedics, Ruijin Hospital, Shanghai Jiao Tong University School of Medicine, Shanghai 200025, P. R. China.

## Abstract

Early and accurate diagnosis of acute kidney injury (AKI) is crucial for clinical treatment. However, most existing fluorescent probes are prone to background interference and limited renal targeting. We developed a ratiometric nanoprobe (RP-SC) that synergistically responded to H_2_O_2_ and targeted kidney injury molecule-1 (KIM-1). In AKI kidneys, RP-SC was hydrolyzed and slowly released encapsulated Hcy-BOH and Cy-Dopa. The H_2_O_2_ level was semi-quantitatively analyzed by fluorescence ratio (*F*_Hcy-BOH_/*F*_Cy-Dopa_). RP-SC exhibited prominent renal fluorescence in AKI mice, making it possible to accurately diagnose AKI and dynamically monitor renal function for up to 60 hours. RP-SC could monitor the process of AKI in vivo and provide an earlier warning of AKI than traditional strategies, subsequently evaluating the recovery of renal function after *N*-acetyl cysteine (NAC) treatment. Our study confirmed the ability of RP-SC for longitudinal monitoring of renal function in vivo by dual-targeting capacity, providing an effective tool for accurate diagnosis of early AKI while tracking the dynamic process of AKI treatment.

## Teaser

A renal-targeting ratiometric nanoprobe responds to H_2_O_2_ for acute kidney injury early diagnosis and disease tracking.

## INTRODUCTION

Acute kidney injury (AKI) is a clinical syndrome defined as a rapid deterioration of renal function, with high incidence and mortality rates (*1*). AKI can be caused by a variety of factors, including tumor, trauma, and drug toxicity such as the anticancer drug cisplatin and aminoglycoside antibiotics (*2*). Early and accurate diagnosis followed by timely protective interventions is an effective approach to prevent AKI. The clinical diagnosis of AKI mainly relies on the measurement of serum creatinine (SCr) and blood urea nitrogen (BUN) in patients, whose levels only change significantly when renal function is severely lost (glomerular filtration rate ≤ 50%), making them failing for early diagnosis of AKI (*3*). Although traditional imaging diagnostics, such as computed tomography (CT), magnetic resonance imaging (MRI), and ultrasound can noninvasively detect anatomical and histological changes in the kidneys, they lack the sensitivity to diagnose early AKI at the molecular level (*4*). Starting from the early molecular mechanisms of kidney injury, the identification and targeting of key signaling molecules are crucial for the early diagnosis of AKI.

Oxidative stress is regarded as a key pathogenic factor in AKI (*5*). Disruption of the redox balance within damaged renal cells leads to the accumulation of reactive oxygen species (ROS), which induces renal cell apoptosis and tissue inflammation (*6*). Numerous studies have shown that ROS levels in the kidney increase significantly in the early stage of AKI caused by ischemia and drug toxicity (*7*, *8*). As a hub of ROS transformation in vivo, H_2_O_2_ can provide more specific and detectable oxidative signals to reflect the redox status of the kidneys than other ROS with higher reactivity and shorter life span, thus being regarded as a sensitive biomarker for early AKI (*9*–*11*). Owing to its relative stability and diffusibility, H_2_O_2_ rapidly accumulates in injured renal regions to induce lipid peroxidation, protein oxidation, and DNA damage, ultimately leading to the damage of renal tubular epithelial cells (*12*, *13*). After injury and regeneration, kidney injury molecule-1 (KIM-1) is highly expressed and participates in the regulation of immune responses, whereas normal renal tubular epithelial cells hardly express KIM-1 (*14*, *15*). Therefore, intracellular H_2_O_2_ and KIM-1 can be considered as two typical biomarkers for early AKI, reflecting the pathological features from the perspectives of oxidative stress and structural damage, respectively. Developing detection methods that simultaneously target both two biomarkers could not only enable early molecular diagnosis of AKI but also provide deeper insights into its pathogenesis.

Recently, fluorescent imaging has played an important role in AKI research due to its noninvasive, high sensitivity, and real-time imaging capabilities (*16*–*18*). Many fluorescent probes targeting key biomarkers of AKI, such as phosphatidylserine, KIM-1, NAG, GGT, O_2_^·-^, and HOCl—have been designed (*19*). For example, Pu and Huang (*20*, *21*) have developed a series of efficient renal-clearance probes functionalized with (2-hydroxypropyl)-β-cyclodextrin for real-time imaging of drug-induced AKI; Weng and co-workers (*22*) have reported probes targeting phosphatidylserine and caspase-3 for diagnosing early AKI; Tian *et al.* have synthesized fluorescent/photoacoustic probes activated by ROS for early AKI detection (*23*, *24*); Zhang and colleagues (*25*) have designed a supramolecular fluorescent probe based on cyanine for high-contrast imaging of early AKI. However, the diagnostic mechanism of most of the developed probes relied on the fluorescence activation upon recognition of AKI-related molecular markers. The relative fluorescence intensity may be influenced by probe diffusion and imaging background, leading to potential false positives and affecting the accurate early diagnosis of AKI. Moreover, some probes exhibit limited renal targeting, complicating the distinction between renal imaging signals and surrounding tissue background. These probes were rapidly cleared from the kidneys, making it difficult to monitor changes in renal function during AKI progression. Ratiometric imaging collects signals from two separate fluorescence channels and calculates their ratio, which could effectively eliminate interference from uneven probe distribution, fluctuations in excitation intensity, and tissue thickness, thereby markedly enhancing the reliability and reproducibility of imaging. In addition, the ratiometric signal directly corresponds to pathology-related biomarker levels, enabling quantitative or semi-quantitative monitoring of biomarkers with higher sensitivity and specificity, thus providing previously unexplored possibilities for high specificity renal imaging (*26*, *27*).

**Fig. 1.**
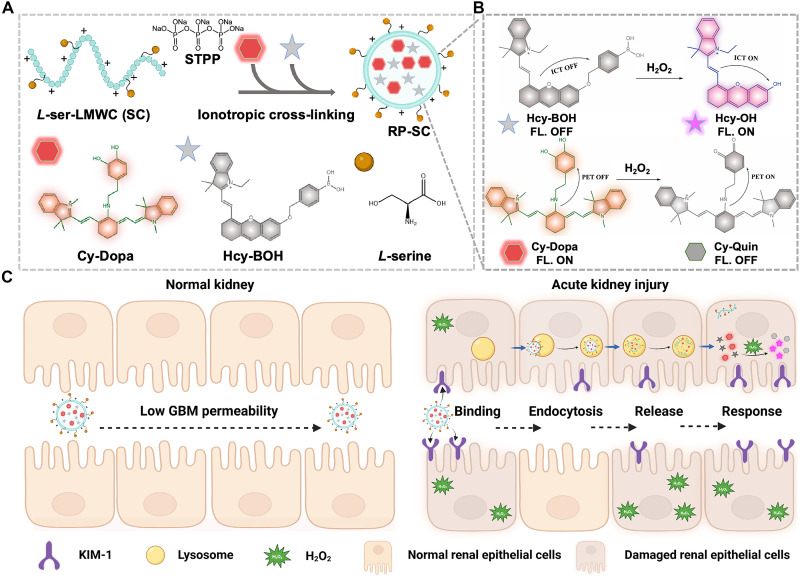
The schematic diagram of RP-SC synthesis and action modes. (**A**) Schematic diagram of synthesizing RP-SC and (**B**) the reaction mechanism of Hcy-BOH and Cy-Dopa with H_2_O_2_. (**C**) Mechanism of RP-SC synergistically responding to H_2_O_2_ and targeting KIM-1 for the diagnosis of early AKI. This figure was created by “BioRender.com.” [license number: ZF28P9NTM7. Created in BioRender. Z. Liu (2025); https://BioRender.com/sxydw7p].

Here, we reported an activable ratiometric nanoprobe named Ratiometric Probe–l-serine-modified low molecular weight chitosan (RP-SC) by coating two H_2_O_2_-specific fluorescence sensors Hcy-BOH and Cy-Dopa with l-serine–modified low–molecular weight chitosan (SC). In response to H_2_O_2_, the fluorescence intensity changes of Hcy-BOH and Cy-Dopa exhibited opposite trends, allowing their ratio to sensitively detect H_2_O_2_ levels. Meanwhile, the l-serine on the SC carrier can specifically bind to the overexpressed KIM-1 in the AKI kidneys. In this study, we demonstrated that the designed RP-SC could synergistically respond to H_2_O_2_ and target KIM-1 for early diagnosis of cisplatin-induced AKI. With an appropriate particle size (53.3 ± 1.0 nm) and zeta potential (+31.7 mV), RP-SC could efficiently penetrate the glomerular basement membrane (GBM) of damaged renal epithelial cells and target regions with KIM-1 protein overexpression, followed by controlled release of two sensors Hcy-BOH and Cy-Dopa. Sensitive ratiometric imaging enabled RP-SC to distinguish H_2_O_2_ levels in live cells and AKI kidneys, thereby detecting the degree and process of AKI in vivo. In contrast, RP-SC struggled to penetrate the GBM of normal kidneys and showed minimal response to low levels of H_2_O_2_. RP-SC also showed excellent performance for continuous monitoring of renal function in AKI mice (up to 60 hours) and for accurately evaluating the therapeutic effect of anti-AKI drug of *N*-acetyl cysteine (NAC) on AKI mice. Furthermore, we demonstrated that RP-SC could detect the occurrence of early AKI at 12 hours after cisplatin induction, significantly earlier than that determined by blood biomarkers and histopathology, highlighting the potential for early AKI diagnosis (Fig. 1).

## RESULTS

### Design and synthesis of RP-SC

Based on the pathogenic factors of early AKI and the development prospect of nanomedicine (*28*), we designed a nanoprobe named RP-SC synergistically responding to H_2_O_2_ and targeting KIM-1 to achieve target-enabled activation and retention effect for improving AKI imaging. Given the relatively deep location of the kidney in vivo, fluorescence imaging with strong tissue penetration and high brightness is necessary. Therefore, two near-infrared (NIR) fluorescent dyes cyanine and semi-cyanine with good biocompatibility were used as fluorophores (*29*). After functionalizing them with dopamine and phenylboric acid groups, two H_2_O_2_-sensitive fluorescence sensors Cy-Dopa and Hcy-BOH were obtained. After Cy-Dopa recognized H_2_O_2_, the phenolic hydroxyl group of dopamine was oxidized to its quinone form (Cy-Quin), triggering photoinduced electron transfer (PET) and resulting in fluorescence quenching. For Hcy-BOH, upon recognition of H_2_O_2_, the phenylboric acid group was removed to release the free Hcy-OH, accompanied by fluorescence enhancement due to the restoration of the intramolecular charge transfer (ICT) effect. By detecting the fluorescence ratio of these two sensors (*F*_Hcy-BOH_/*F*_Cy-Dopa_), the H_2_O_2_ levels in AKI kidneys could be sensitively monitored. However, similar to many small-molecule sensors, Cy-Dopa and Hcy-BOH tended to accumulate in the reticuloendothelial system such as the liver and spleen, affecting their distribution in the kidney. Coating with positively charged and water-soluble delivery carriers is a general approach to improving the renal distribution of Cy-Dopa and Hcy-BOH, facilitating their penetration through the negatively charged GBM into the kidneys (*30*).

Low–molecular weight chitosan (LWMC) is a natural cationic polysaccharide with a p*K*_a_ (where *K*_a_ is the acid dissociation constant) of about 6.5 to 7.0 and with excellent biocompatibility and biodegradation, which makes it a promising renal-targeted drug carrier (*31*). The LMWC probes could bind to the megalin receptors present in renal tubular epithelial cells and be selectively internalized into the lysosome through megalin receptor–mediated endocytosis (*32*). Subsequently, LMWC in the designed RP-SC slowly degrades and releases the encapsulated Cy-Dopa and Hcy-BOH in AKI kidney, effectively prolongating the retention time of the two sensors in the kidney (*33*). In addition, LMWC has abundant reaction sites, which can be modified with l-serine to specifically bind to KIM-1, which is overexpressed in damaged renal epithelial cells (*34*). We prepared l-serine–modified LMWC (SC) via a simple condensation reaction and characterized it by ^1^H NMR, confirming that l-serine was effectively modified at a molar ratio of 2:1 (l-serine: LMWC). Using an ionic cross-linking technique (*35*), the designed RP-SC has been successfully prepared by coating Cy-Dopa and Hcy-BOH with SC (mass ratio: 1.1:1:20). After dialysis purification, the encapsulation efficiency of RP-SC was 81.99 ± 1.43%, and loading content was 12.23 ± 0.25% by high-performance liquid chromatography (HPLC) and ultraviolet-visible (UV-Vis) spectroscopy.

### Physicochemical properties of RP-SC

**Fig. 2.**
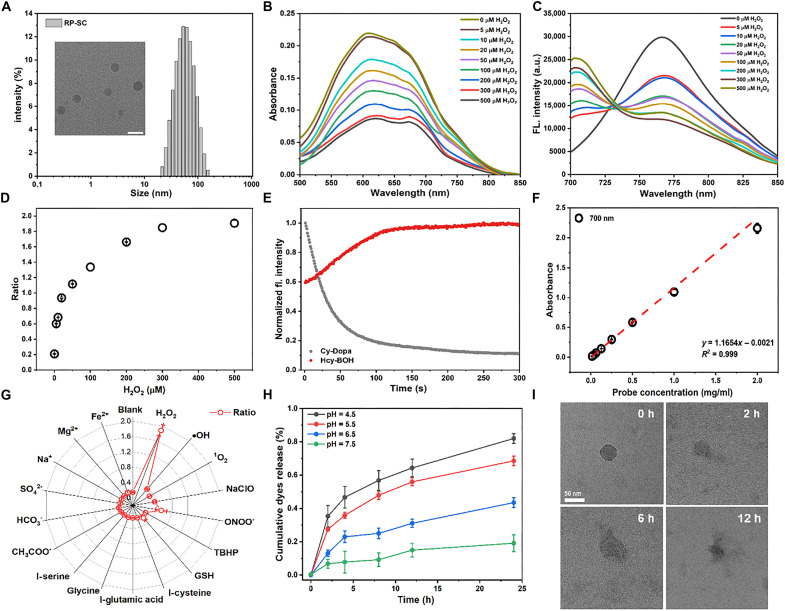
Physicochemical properties of RP-SC. (**A**) DLS measurement and TEM image of RP-SC in PBS. Scale bar, 50 nm. (**B**) Absorption spectra of RP-SC (0.1 mg/ml) in the absence or presence of H_2_O_2_ with different concentrations. (**C**) Fluorescence spectra of RP-SC (0.1 mg/ml) in the absence or presence of H_2_O_2_ with different concentrations. (**D**) Dose-response curve of RP-SC (0.1 mg/ml) to H_2_O_2_ (0 to 500 μM). (**E**) Time-dependent response of Hcy-BOH (5.0 μM) and Cy-Dopa (5.0 μM) to 100 μM H_2_O_2_. (**F**) Absorbance at 700 nm versus the concentrations of RP-SC (0 to 2.0 mg/ml) in PBS solution (0.10 M, pH = 7.4). (**G**) Ratiometric fluorescence responses of RP-SC to H_2_O_2_ (500 μM) and various potential interferences (200 μM ·OH; 200 μM ^1^O_2_, 200 μM NaClO, 200 μM ONOO^−^, 100 μM TBHP, 1.0 mM GSH, 100 μM l-cysteine, 100 μM l-glutamic acid, 100 μM l-glycine, 100 μM l-serine, 100 μM CH_3_COO^−^, 100 μM HCO_3_^−^, 100 μM SO_4_^2−^, 100 μM Na^+^, 100 μM Mg^2+^, and 100 μM Fe^2+^. (**H**) Percentage release of RP-SC (1.0 mg/ml) in different pH conditions. (**I**) TEM images of RP-SC in PBS buffer (pH 5.0) at different time points. Scale bar, 50 nm. a.u., arbitrary unit.

First, the reactivity of Cy-Dopa and Hcy-BOH to H_2_O_2_ was investigated in phosphate-buffered saline (PBS). Cy-Dopa exhibited a prominent absorption band centered at 623 nm, accompanied by a NIR fluorescence maximum at 766 nm. Upon addition of H_2_O_2_, UV-Vis spectrum showed that the absorption peak of Cy-Dopa at 623 nm gradually decreased while a new peak at 532 nm emerged, indicating the product of Cy-Quin. Correspondingly, the NIR fluorescence of Cy-Dopa at 766 nm was effectively quenched, indicating the formation of PET effect. After the addition of 200 μM H_2_O_2_, the fluorescence intensity of Cy-Dopa decreased to ^1^/_10_ of the initial level, and the fluorescence quantum yield decreased from 0.147 to 0.011 (fig. S1). For Hcy-BOH, the absorption peaks at 618 and 678 nm in the UV-Vis spectrum of Hcy-BOH showed an inversion in intensity after the addition of H_2_O_2_. And the NIR fluorescence at 706 nm enhanced with increasing concentration of H2O2, which indicated the successful release of free Hcy-OH. The fluorescence intensity enhanced 16.9-fold after adding 500 μM H_2_O_2_, and the quantum yield increased from 0.0023 to 0.176, attributed to the strong ICT in Hcy-OH (fig. S3). The fast reaction kinetics further demonstrated that both sensors were sensitive to H_2_O_2_, with reactions completing in ~100 s after the addition of 100 μM H_2_O_2_ (Fig. 2E). Meanwhile, the water solubility of Cy-Dopa and Hcy-BOH was also verified, with both being uniformly dispersed in PBS at concentrations of 50 and 20 μM, respectively. We also tested the stability of Cy-Dopa and Hcy-BOH in a range of pH buffer solutions, and the results showed that the fluorescence of both sensors remained stable under physiological conditions (figs. S2 and S4). The photostability test demonstrated that both sensors exhibited good stability (fig. S5).

Inspired by the sensitive response of Cy-Dopa and Hcy-BOH to H_2_O_2_, the physicochemical properties of RP-SC were tested. The RP-SC exhibited a uniformly dispersed, spherical morphology measuring 45 nm, on average, as observed through transmission electron microscopy (TEM) (Fig. 2A). Dynamic light scattering (DLS) results showed that the hydrodynamic diameter of RP-SC was about 53.3 ± 1.0 nm, attributed to the presence of hydration layers. The zeta potential of RP-SC was +31.7 mV, which corresponded to the abundant positive charge from LMWC (fig. S6). The appropriate particle size (<100 nm) and positive zeta potential facilitated RP-SC to penetrate the negative glomerular filtration barrier. Subsequently, we investigated the response performance of RP-SC to H_2_O_2_. As shown in Fig. 2B, the absorbance of the broad peak gradually decreased after adding H_2_O_2_. Initially, RP-SC only displayed an obvious NIR fluorescence emission at 766 nm from Cy-Dopa. Upon reacting with H_2_O_2_ (0 to 500 μM), RP-SC exhibited a remarkable decrease in fluorescence at 766 nm, accompanied by increased fluorescence at 706 nm from Hcy-OH with isosbestic points of about 730 nm. The well-resolved fluorescence spectra for Cy-Dopa and Hcy-BOH fluorophores (Δλ = 60 nm) efficiently minimized the spectral cross-talk and improved the dynamic range of ratiometric imaging (Fig. 2, C and D). It is worth noting that the SC coating did not significantly affect the sensitivity of Hcy-BOH and Cy-Dopa to H_2_O_2_.

The RP-SC exhibited good water solubility and was not significantly polymerized even at a high concentration (2.0 mg/ml) (Fig. 2F). In addition, the fluorescence response of RP-SC to other interferents (including other kinds of ROS and amino acids and various ions) was negligible at their physiological concentrations (Fig. 2G). In vitro release behaviors of RP-SC were investigated using the dialysis method at different pH levels (*36*). The results showed that RP-SC remained stable over a 24 hours incubation period at pH 7.5, whereas in acidic environments (pH = 4.5 to 6.5), the release rate was significantly accelerated. In a solution with pH = 4.5, which is close to the pH of the lysosome, the Cy-Dopa and Hcy-BOH were released at a nearly 1:1 molar ratio, with a cumulative release of 64.3 ± 5.3% after incubated for 12 hours (Fig. 2H). Moreover, TEM results also indicated that the structure of RP-SC gradually collapsed in the acidic environment, and its spherical morphology disappeared (Fig. 2I).

### Ratiometric imaging of RP-SC in living HK-2 cells

**Fig. 3.**
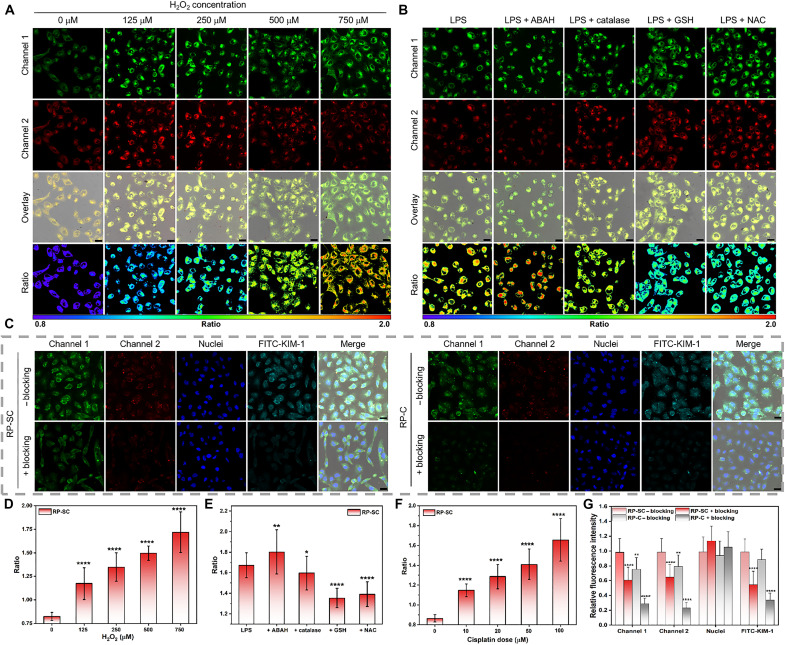
Ratiometric imaging of RP-SC in living HK-2 cells. (**A**) Ratiometric fluorescence images of living HK-2 cells treated with different concentrations of H_2_O_2_ (0 to 750 μM) and then loaded with RP-SC (100 μg/ml) for 4 hours. Channel 1: 680 to 720 nm; channel 2: 750 to 850 nm. Scale bar, 25 μm. (**B**) Ratiometric fluorescence images of endogenous H_2_O_2_ in living HK-2 cells. The cells were pretreated with LPS (2.0 μg/ml) for 24 hours and then treated with 100 μM ABAH for 6 hours before RP-SC loading. For other groups, cells were treated with LPS (2.0 μg/ml) for 24 hours and then incubated with catalase (1.0 mg/ml), 1.0 mM GSH, or 1.0 mM NAC for 24 hours before RP-SC loading, respectively. Scale bar, 25 μm. (**C**) Confocal images of HK-2 cells that were previously stimulated with cisplatin for 24 hours and then treated with RP-SC (100 μg/ml) or RP-C for another 4 hours. “+ blocking” indicated the cells were pretreated with KIM-1 (30 μg/ml) antibody for 1 hour before administration of RP-SC or RP-C. “− blocking” indicated without treatment of KIM-1 antibody. Nuclei stained with 4′,6-diamidino-2-phenylindole (DAPI) were shown in blue. Green indicated immunostaining for KIM-1. (**D**) Ratio of living HK-2 cells treated with different concentrations of H_2_O_2_. (**E**) Ratio of endogenous H_2_O_2_ in living HK-2 cells. (**F**) Ratio of living HK-2 cells treated with different doses of cisplatin. (**G**) Quantification of fluorescence images in different channels shown in (C). All data were presented as means ± SD. Statistical differences between groups were labeled with asterisks, *P < 0.05, **P < 0.01, and ****P < 0.0001 by Student’s *t* test.

After confirming the good biocompatibility of RP-SC by MTT [(*3*-(*4*,*5*-dimethylthiazol-*2*-yl)-*2*,*5*-diphenyltetrazolium bromide)] assay (fig. S7), we explored its imaging potential in human proximal tubular cells, HK-2 cells. We studied the subcellular distribution of RP-SC in cells by costaining imaging and confirmed that RP-SC colocalized well with LysoTracker Green, indicating that it could selectively gather in lysosome (fig. S8). Subsequently, the response of RP-SC to H_2_O_2_ in living cells was validated. HK-2 cells were treated with H_2_O_2_ at various doses (0, 125, 250, 500, and 750 μM) for 2 hours and then stained with RP-SC (100 μg/ml) for 4 hours before imaging. HK-2 cells stained with only RP-SC showed weak fluorescence in both channel 1 (680 to 720 nm) and channel 2 (750 to 850 nm), suggesting minimal probe uptake and low endogenous H_2_O_2_ levels. However, HK-2 cells treated with H_2_O_2_ exhibited significantly enhanced fluorescence in both two channels, with obvious relative intensity difference. The fluorescence ratio (*R*_cell_ = *F*_channel 1_/*F*_channel 2_) gradually increased with the H_2_O_2_ concentration. Compared with 0.82 ± 0.04 in cells treated without H_2_O_2_, the *R*_cell_ increased to 1.17 ± 0.17, 1.34 ± 0.15, 1.50 ± 0.08, and 1.72 ± 0.22 in cells treated with 125, 250, 500, and 750 μM H_2_O_2_, respectively (Fig. 3, A and D). Moreover, the ability of RP-SC to detect endogenous H_2_O_2_ was also verified. After induction with lipopolysaccharide (LPS; 2.0 μg/ml) for 24 hours, HK-2 cells were incubated with RP-SC for confocal imaging. Confocal imaging results revealed strong fluorescence in channel 1, while fluorescence in channel 2 was weaker, with a high *R*_cell_ (1.67 ± 0.12) indicating elevated levels of endogenous H_2_O_2_. We also added ABAH, a myeloperoxidase inhibitor to stimulate H_2_O_2_ levels (*37*), and the corresponding *R*_cell_ increased to 1.80 ± 0.22. After treated with catalase, the *R*_cell_ decreased to 1.56 ± 0.16. Similarly, when we treated the cells with reductive glutathione (GSH) and NAC, the *R*_cell_ decreased to 1.35 ± 0.09 and 1.39 ± 0.12, respectively, indicating that H_2_O_2_ was effectively eliminated. These results demonstrated that RP-SC could sensitively monitor fluctuations in H_2_O_2_ levels in cells (Fig. 3, B and E).

In addition, cellular damage was induced with cisplatin, a classic anticancer drug known to be nephrotoxic (*38*). After treating HK-2 cells with a range of cisplatin concentrations (0 to 100 μM) for 24 hours, the RP-SC was added (fig. S9). Imaging results showed that cells treated with cisplatin exhibited strong fluorescence. With the increase of cisplatin dose, the *R*_cell_ increased from 0.86 ± 0.03 (0 μM) to 1.15 ± 0.07 (10 μM), 1.28 ± 0.12 (20 μM), 1.41 ± 0.15 (50 μM), and 1.66 ± 0.21 (100 μM), indicating elevated intracellular H_2_O_2_ levels (Fig. 3F and fig. S9). KIM-1 expression was also up-regulated in the cisplatin-induced cells, which could be confirmed by Western blotting assay (fig. S10). To investigate the high uptake of RP-SC by damaged renal cells, cisplatin-stimulated HK-2 cells pretreated with or without the blocking agent of KIM-1 antibody were incubated with RP-SC for 4 hours. Compared to cells un-incubated by KIM-1 antibody (− blocking), cells treated with KIM-1 antibody (+ blocking) displayed significantly reduced fluorescence, indicating a lower RP-SC uptake. In the − blocking cells, the fluorescence of RP-SC showed good colocalization with that of fluorescein isothiocyanate (FITC)–KIM-1. These results suggested that the specific binding of SC to KIM-1 facilitated the internalization of RP-SC by cells, as reported in previous studies (*34*). We also prepared RP-C, a control probe consisting of chitosan-coated Hcy-BOH and Cy-Dopa only, and coincubated it with cells. Fluorescence imaging showed that cells stained with RP-C exhibited weaker fluorescence than those treated with RP-SC, indicating less uptake of RP-C without l-serine modification by the cells. After KIM-1 antibody treatment, the intracellular fluorescence of RP-C was more barren, further confirming that the up-regulation of KIM-1 in damaged renal cells promoted the cell internalization of l-serine through the specific interaction between KIM-1 and l-serine (Fig. 3, C and G).

### Ratiometric imaging of AKI mice in vivo

**Fig. 4.**
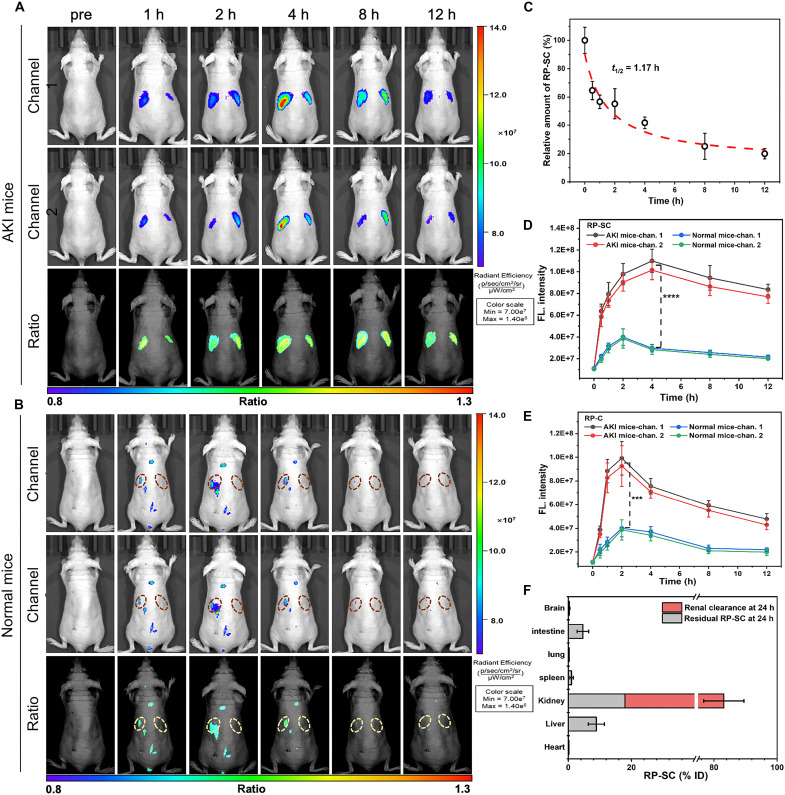
Ratiometric imaging of AKI mice in vivo. Ratiometric fluorescence images of AKI mice (**A**) or normal mice (**B**) prior (pre) or at 1, 2, 4, 8, and 12 hours post-intravenous injection of RP-SC (1.0 mg/ml, 100 μl). Channel 1: 700 to 720 nm; channel 2: 760 to 780 nm. (**C**) Relative amount of RP-SC (%) remained in the blood at 0, 0.5, 1, 2, 4, 8, and 12 hours after intravenous injection of RP-SC (1.0 mg/ml, 100 μl) into healthy mice. (**D**) Quantification of fluorescence images of AKI mice and normal mice over time after intravenous injection RP-SC. (**E**) Quantification of fluorescence images of AKI mice and normal mice over time after intravenous injection RP-C. (**F**) HPLC quantitative analysis of residual RP-SC in major organs (gray bar) and RP-SC excreted from kidney into urine (red bar) 24 hours after intravenous injection. All data are presented as means ± SD. Statistical differences between groups are labeled with asterisks, ***P < 0.001 and ****P < 0.0001 by Student’s *t* test.

Encouraged by the intriguing photophysical properties and reliable performance of RP-SC in cells, we further investigated its imaging performance in vivo. Hemolysis assays and pathological section analysis confirmed the excellent biocompatibility of RP-SC (figs. S11 and S12). The RP-SC was injected intravenously into normal mice and cisplatin-induced AKI mice, followed by continuous imaging. Dorsal whole-body fluorescence imaging revealed notable differences in fluorescence distribution and intensity between normal and AKI mice (Fig. 4, A and B). After 1-hour injection of probe, obvious fluorescence appeared in both two channels in the kidney regions of AKI mice and reached the maximum after 4 hours, with an enhancement of about 40%. The fluorescence gradually decreased over time, which was attributed to the excretion of the probe into the urine via the kidneys (fig. S13). Notably, the renal fluorescence ratio (*R*_kidney_ = *F*_channel 1_/*F*_channel 2_) remained nearly unaffected by metabolism, stabilizing at ~1.09 ± 0.03, indicating the excellent stability of ratiometric imaging in vivo. In contrast, the fluorescence distribution in normal mice was almost nonspecific, and the fluorescence in the kidney regions was poor, corresponding to few probes being excreted into the urine (fig. S14). After 4 hours postinjection, the renal fluorescence intensity of AKI mice was ~3.7-fold higher than that in normal mice (Fig. 4D). The substantial difference in renal fluorescence may be due to the ability of RP-SC to penetrate GBM and the varying ROS levels in the kidney. The RP-SC could easily penetrate the GBM in AKI mice and specifically bind to the overexpressed KIM-1, achieving kidney retention and responding to excessive H_2_O_2_. However, RP-SC struggled to penetrate the undamaged GBM (pore size was about 2 to 8 nm) (*39*) and showed minimal response to low concentrations of H_2_O_2_ in normal mice. The blood half-life (*t*_1/2_) of RP-SC in healthy mice was 1.17 hours, indicating that it could rapidly clear from the blood circulation (Fig. 4C). The renal sections of AKI mice were collected at 4 hours after administration of RP-SC, and their microscopic fluorescence images were then captured. As shown in fig. S15, outstanding colocalization of KIM-1 and RP-SC was observed, confirming the probe’s specific targeting of KIM-1. In vitro imaging of organs from AKI and normal mice demonstrated that the fluorescence in AKI kidneys was significantly stronger than in other organs, indicating selective renal uptake. The renal fluorescence of normal mice was only 25% of that in AKI mice (fig. S16). The quantitative analysis of tissue homogenates showed that 18% of RP-SC remained in the kidneys of AKI mice 24 hours after injection, while ~65 ± 6.4% was excreted into urine, demonstrating the probe’s effective renal targeting and retention, providing the possibility for long-term imaging (Fig. 4F).

Photopenetration is one of the crucial concerns in the fluorescence imaging of living animals. To verify the tissue penetration ability of RP-SC, the renal regions of AKI mice were covered with sliced chicken breast tissues of varied thicknesses (0 to 3.0 mm) and monitored the fluorescence. Although both *F*_channel 1_ and *F*_channel 2_ gradually decreased with the increasing tissue thickness, their ratio kept stable across a wide range of thicknesses (fig. S17). We have also further evaluated the potential influence of the experimental setups, including the excitation/emission wavelength and distance between the excitation light and the mice. Although the selection of excitation and emission wavelength may lead to variations in *F*_channel 1_, *F*_channel 2_, and their ratio, the selected wavelength ranges (λ_ex_ = 675 nm; channel 1: λ_em_ = 700 to 720 nm; channel 2: λ_em_ = 760 to 780 nm) provided a broad and reliable dynamic range (fig. S18). The *F*_channel 1_ and *F*_channel 2_ increased with the decreased distance between mice and excitation light due to the improved light intensity on the mice. However, their ratio remained nearly constant with the distance (fig. S19).

In addition, the imaging performance of the control probe RP-C was also explored in vivo. The imaging results showed that obvious fluorescence appeared in the kidney regions 1 hour after RP-C injection, weakened with time after reaching the maximum at 2 hours, and partially disappeared after 12 hours with only 57% of that of RP-SC (Fig. 4E). Similarly, RP-C exhibited nonspecific fluorescence in normal mice, indicating poor renal uptake (fig. S20). In vitro organ imaging revealed that 12 hours after RP-C injection, the renal fluorescence in AKI mice was stronger than other organs but significantly weaker than that of RP-SC. In normal mice, the fluorescence signals in the kidney were almost undetectable (fig. S21). These results demonstrated that RP-SC exhibited superior renal targeting capability and high sensitivity in diagnosing AKI mice than that of RP-C mainly due to the l-serine modifications.

**Fig. 5.**
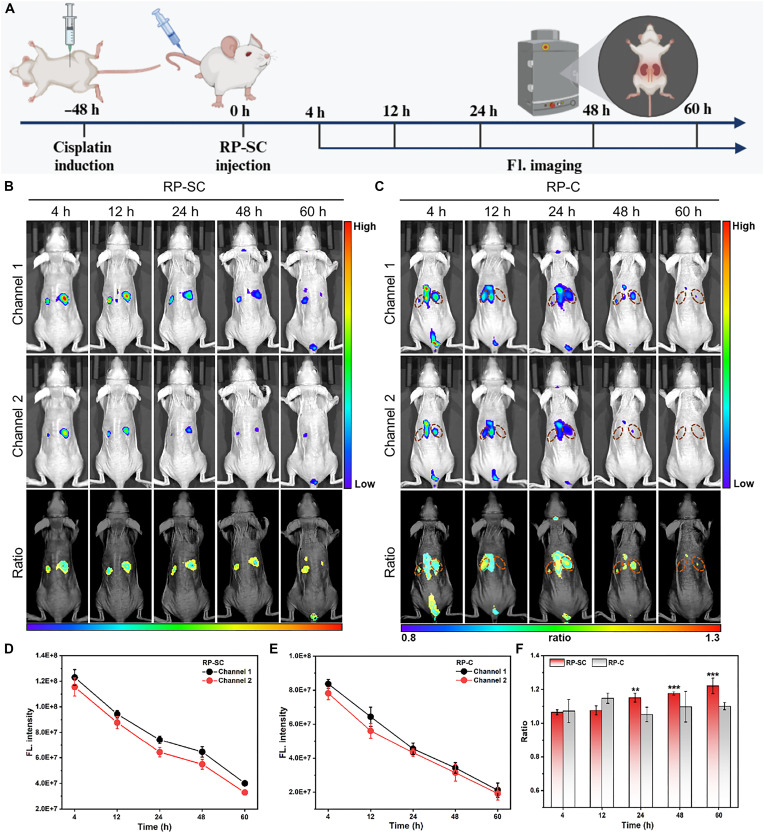
Dynamic monitoring of renal function in AKI mice in vivo. (**A**) Schematic illustration of the experimental protocol. This figure was created by BioRender.com. [license number: RZ28P9OF0X. Created in BioRender. Z. Liu (2025); https://BioRender.com/95hw1nj]. Ratiometric fluorescence images of AKI mice at 4, 12, 24, 48, and 60 hours post-intravenous injection of RP-SC (**B**) and RP-C (**C**) (1.0 mg/ml, 100 μl). Channel 1: 700 to 720 nm; channel 2: 760 to 780 nm. (**D** and **E**) Quantification of fluorescence images of AKI mice over time after intravenous injection RP-SC or RP-C. (**F**) Ratio of kidneys of AKI mice over time after intravenous injection RP-SC or RP-C.

Given the rapid progression of AKI, dynamically monitoring renal function in AKI mice is crucial for diagnosis (Fig. 5A). Therefore, we longitudinally compared renal fluorescence at different time points after RP-SC injection. Through dynamic imaging, we found that RP-SC sensitively detected gradually increased *R*_kidney_ of AKI mice, indicating an intensification of oxidative stress in the kidneys. Even 60 hours postinjection, the renal fluorescence could still be distinguished from the surrounding tissue (Fig. 5, B and D). Because of the appropriate size and positive charge properties, RP-SC was mainly metabolized in a relatively single metabolic pathway through the kidneys. Besides, the complete GBM of normal nephrons have a lower filtration rate for RP-SC, which ensured the long-term residence of RP-SC in the circulation. While in damaged nephrons, the l-serine on the surface of RP-SC can specifically bind to KIM-1 on the surface of the damaged renal epithelial cells. During renal metabolism, a considerable portion of RP-SC will remain inside the kidney, causing long-term renal retention without being directly metabolized. Thus, RP-SC can remain in the circulatory system for a long time while accumulating in damaged kidneys. However, RP-C failed to accurately diagnose fluctuations in renal redox states with low signal-to-noise, and fluorescence signals were lost over time (Fig. 5, C and E). All results indicated that RP-SC could not only enable precise AKI diagnosis but also allow for long-term dynamic monitoring renal function in AKI mice (Fig. 5F).

### Detection of the degree and process of AKI in vivo

**Fig. 6.**
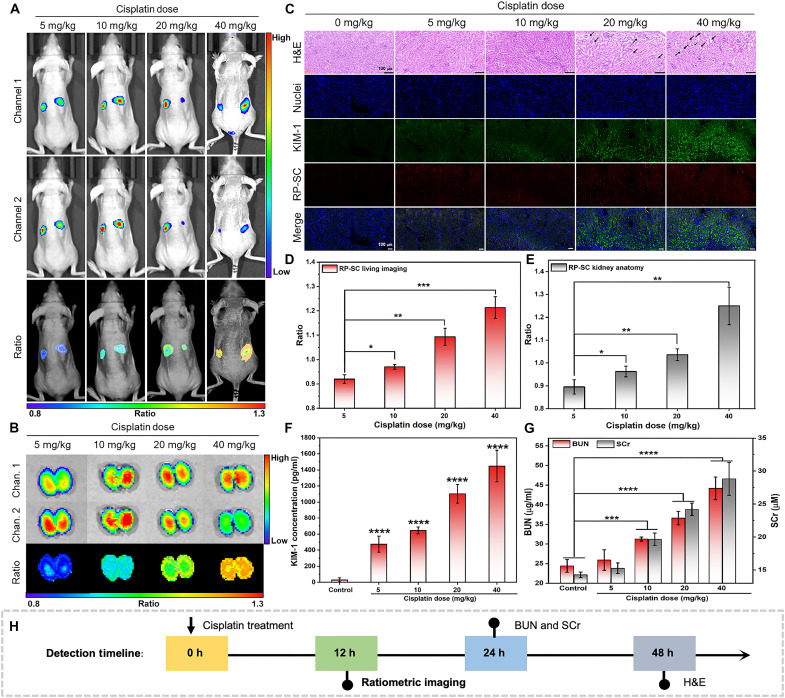
Detection of the degree and process of AKI in vivo. Ratiometric fluorescence images of mice (**A**) and anatomical kidneys (**B**) receiving intraperitoneal injection of different doses of cisplatin for 48 hours, followed by intravenous injection of RP-SC (1.0 mg/ml, 100 μl) at 4 hours. Channel 1: 700 to 720 nm; channel 2: 760 to 780 nm. (**C**) H&E staining and immunofluorescence staining of kidney tissue slices resected from mice posttreatment with different doses of cisplatin. Scale bar, 100 μm. (**D** and **E**) Ratio of mice and anatomical kidneys shown in (A) and (B). (**F**) Concentrations of KIM-1 in kidney homogenates of mice treated with different doses of cisplatin. (**G**) Concentrations of BUN and SCr in serum of mice treated with different doses of cisplatin. All data are presented as means ± SD. Statistical differences between groups are labeled with asterisks, *P < 0.05, **P < 0.01, ***P < 0.001, and ****P < 0.0001 by Student’s *t* test.

After validating the excellent performance of RP-SC in accurately diagnosing AKI, we applied it for detecting the degree of AKI in vivo. A series of doses (0 to 40 mg/kg) of cisplatin were intraperitoneally injected into mice to induce varying degrees of AKI, and RP-SC was intravenously injected for imaging. As shown in Fig. 6A, the kidney regions of AKI mice exhibited prominent fluorescence signals, with the *R*_kidney_ increasing with the dose of cisplatin, indicating elevated H_2_O_2_ levels in the kidneys (Fig. 6D). For mice induced with cisplatin (5 mg/kg), *R*_kidney_ was 0.92 ± 0.02, which increased to 0.97 ± 0.01 (10 mg/kg), 1.09 ± 0.04 (20 mg/kg), and 1.21 ± 0.04 (40 mg/kg). Moreover, the anatomical imaging of the kidneys revealed similar results: The *R*_kidney_ of mice induced with cisplatin (5 mg/kg) was 0.90 ± 0.03, 0.93 ± 0.02 for 10 mg/kg, 1.04 ± 0.03 for 20 mg/kg, and 1.25 ± 0.07 for 40 mg/kg (Fig. 6, B and E). To confirm that the gradual enhanced *R*_kidney_ was correlated to the degree of AKI, we measured SCr and BUN levels in AKI mice to assess renal function. The results indicated that both biomarkers levels significantly increased with the doses of cisplatin (Fig. 6G). For mice induced with cisplatin (40 mg/kg), BUN and SCr levels reached twice that in normal group, suggesting severe renal dysfunction. In addition, hematoxylin and eosin (H&E) staining of kidney tissues showed that tubular cell swelling and necrosis and cast formation indicative of obvious kidney injury appeared after exposure to cisplatin (20 mg/kg). We further performed immunofluorescence staining of KIM-1 in the kidney sections and found that KIM-1 level was deficient in normal kidneys, whereas its expression increased significantly with cisplatin dose in AKI kidneys (Fig. 6C). Enzyme-linked immunosorbent assay results also confirmed a significant up-regulation of KIM-1 in kidney homogenates of AKI mice, with KIM-1 levels in the kidneys of cisplatin-induced mice (40 mg/kg) being 48-fold higher than that without cisplatin treatment (Fig. 6F).

Moreover, RP-SC was also applied to investigate the progression of AKI in mice at 0, 24, 48, and 96 hours posttreatment with cisplatin (20 mg/kg). The results showed that 12 hours after cisplatin induction, the renal fluorescence emerged above the tissue background, with a *R*_kidney_ of 0.85 ± 0.01 and gradually increased to 0.93 ± 0.02 at 24 hours, 1.08 ± 0.02 at 48 hours, and 1.26 ± 0.09 at 96 hours after cisplatin exposure, indicating a sharp rise in H_2_O_2_ levels within the kidneys (fig. S22). In vitro kidney imaging results were also similar, with a *R*_kidney_ of 0.85 ± 0.02 after 12 hours, 0.94 ± 0.01 after 24 hours, 1.06 ± 0.02 after 48 hours, and 1.23 ± 0.12 after 96 hours (fig. S23). H&E staining of kidney tissues showed that tubule morphology appeared normal in the first 24 hours, while cast formation and tubular cell necrosis were observed after 48 hours of cisplatin exposure, indicating significant kidney injury. Immunofluorescence staining revealed KIM-1 expressed at 12 hours after cisplatin induction, with increasing levels of KIM-1 as the cisplatin treatment duration was extended (fig. S24). SCr and BUN results showed that the levels of these biomarkers were significantly elevated at 24 hours posttreatment with cisplatin, increasing to 1.7 times that of normal mice at 48 hours, indicating worsening renal dysfunction (fig. S25). These results affirmed that RP-SC–based ratiometric imaging could provide an earlier warning for the occurrence of early AKI compared to traditional blood biomarkers and tissue H&E staining, making RP-SC a reliable tool for the precise diagnose of early AKI (Fig. 6H).

### Monitoring of therapeutic effect on AKI

**Fig. 7.**
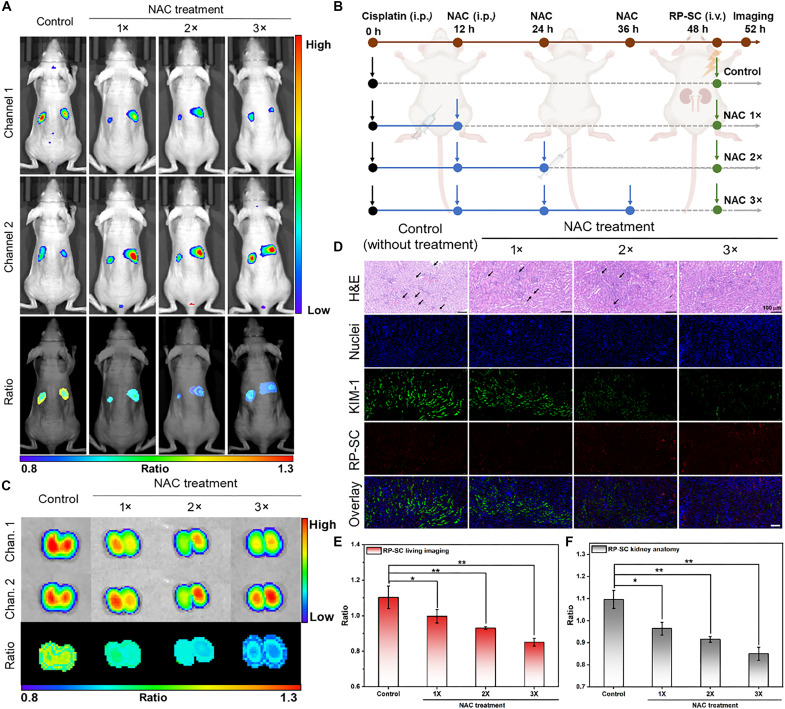
Monitoring of the therapeutic effect on AKI in vivo. Ratiometric fluorescence images of AKI mice (**A**) and anatomical kidneys (**C**) receiving intraperitoneal injection of different doses of NAC, followed by intravenous injection of RP-SC (1.0 mg/ml, 100 μl) at 4 hours. Channel 1: 700 to 720 nm; channel 2: 760 to 780 nm. (**B**) Schematic illustration of the experimental protocol. This figure was created by BioRender.com (license number: GW28P9OZSN. Created in BioRender. Z. Liu (2025); https://BioRender.com/xl2ie6j). The control group was AKI mice without NAC treatment. NAC 1×, 2×, and 3× groups were AKI mice treated with a single dosage (1 × 100 mg/kg, 12 hours), double dosages (2 × 100 mg/kg, 12 and 24 hours), and triple dosages (3 × 100 mg/kg, 12, 24, and 36 hours), respectively. (**D**) H&E staining and immunofluorescence staining of kidney tissue slices resected from mice posttreatment with different doses of NAC. Scale bars, 100 μm. (**E** and **F**) Ratio of AKI mice and anatomical kidneys shown in (A) and (B). All data are presented as means ± SD. Statistical differences between groups are labeled with asterisks, *P < 0.05 and **P < 0.01 by Student’s *t* test. i.p., intraperitoneal; i.v., intravenous.

Timely protective intervention in the early stage of AKI is expected to improve renal function, so evaluating the therapeutic effect of AKI is very important for its comprehensive diagnosis and treatment. NAC, as a classic antioxidant, has been proven to have therapeutic effects on a variety of kidney diseases (*40*). Healthy mice were injected with cisplatin (20 mg/kg) to induce nephrotoxicity and then treated every 12 hours with NAC at a single dose (1×, 12 hours), double dosages (2×, 12 and 24 hours), and triple dosages (3×, 12, 24, and 36 hours), respectively (Fig. 7B). The imaging results showed that compared with the untreated mice (*R*_kidney_ = 1.10 ± 0.06), the *R*_kidney_ of the treated mice was significantly decreased, which was 0.99 ± 0.04 after treated with NAC at a single dose, 0.93 ± 0.01 at double dosages, and 0.85 ± 0.02 at triple dosages (Fig. 7, A and E). In vitro imaging was also consistent with the living imaging findings, with the *R*_kidney_ of the untreated group was reduced from 1.09 ± 0.04 to 0.96 ± 0.03 (1× NAC), 0.92 ± 0.01 (2× NAC), and 0.85 ± 0.03 (3× NAC), respectively, indicating that the oxidative stress of the kidney was effectively ameliorated (Fig. 7, C and F). H&E staining showed that the morphology of tubular was not significantly damaged after 3× NAC treatment, and the expression of KIM-1 was markedly decreased according to the immunofluorescence staining (Fig. 7D). Furthermore, SCr and BUN levels also decreased with increasing NAC dosages. After 3× NAC treatment, SCr and BUN levels were 1.0- and 0.8-fold lower than that in AKI mice without NAC treatment, respectively, approaching the levels observed in the baseline group (fig. S26). These data were consistent with ratiometric fluorescence imaging achieved by RP-SC, confirming the effective recovery of renal function after treatment of AKI mice with 3× NAC. Therefore, the designed RP-SC probe could effectively monitor the therapeutic effects of NAC on AKI and may assist in the development of treatment strategies for AKI through real-time ratiometric imaging.

## DISCUSSION

Based on the typical pathological features of early AKI, including ROS dysregulation and renal cell damage, we developed a ratiometric nanoprobe (RP-SC) that synergistically responded to H_2_O_2_ and targeted KIM-1 to accurately diagnose early AKI. The designed RP-SC could sensitively react with H_2_O_2_, with fast reaction kinetics, excellent selectivity, and stability. We validated that RP-SC could sensitively report fluctuations of H_2_O_2_ levels in HK-2 cells and demonstrated that the specific interaction of SC with KIM-1 facilitated the internalization of RP-SC by renal cells. Outstanding tissue penetration and kidney targeting enable RP-SC to overcome the challenges of in vivo imaging of early AKI. The RP-SC showed obvious fluorescence in kidney regions of cisplatin-induced AKI mice but almost nonspecific distribution in normal mice. With excellent renal retention effect and robust ratiometric imaging capability, RP-SC could dynamically monitor renal function in AKI mice and could be distinguished from surrounding tissue even at 60 hours postinjection. In addition, RP-SC could accurately diagnose different degrees of AKI and monitor the process of AKI. We found that RP-SC provided an earlier warning of the occurrence of early AKI than traditional blood biomarkers and tissue pathology. RP-SC was further applied to evaluate the therapeutic effect of NAC on AKI, confirming that renal function was effectively improved after treatment with three dosages of NAC. This study demonstrates that the designed RP-SC could accurately diagnose early AKI and monitor renal function in vivo, which is also expected to assist in screening prognostic assessment of AKI treatment.

## MATERIALS AND METHODS

### Materials and instruments

For synthesis, general chemical reagents and solvents were purchased from commercial suppliers, including Sigma-Aldrich, Adamas-beta, TCI, Innochem, Energy Chemical, and Aladdin. Unless otherwise stated, all the commercial reagents were used without further purification. All chemicals and solvents used were of analytical grade (purity >99.0%).

Thin-layer chromatography (TLC) was performed on TLC-aluminum sheets (Silica gel 60 F254). Flash column chromatography was performed with General-Reagent silica gel (200 to 300 mesh and type H). ^1^H NMR spectra and ^13^C NMR were recorded on the Bruker Avance spectrometer (400 MHz for ^1^H NMR and 101 MHz for ^13^C NMR) with tetramethylsilane as the internal standard. High-resolution mass spectra were acquired with a Bruker QTOF Impact using electrospray ionization. HPLC purification was performed on a SHIMADZU LC-20A (YMC-Pack ODS-A) system. Eluent A (purified water removes bubbles through ultrasound) and eluent B (acetonitrile containing 0.1% trifluoroacetic acid) were used for HPLC purification. Compounds purified by HPLC were frozen into powder by a bench top freeze dryer. Fluorescence and UV-Vis spectra were recorded on a SHIMADZU RF-6000 and a SHIMADZU UV-2600 spectrophotometer. A BioTek microplate reader was used for the measurement of MTT assay, and some biochemical markers such as BUN, SCr, and KIM-1. The morphology of the nanoprobe was characterized at a Thermo Fisher Scientific TEM operated at 200 kV. The particle size and size distribution were obtained by DLS at 25°C by means of a 90 Plus/BI-MAS equipment. Zeta potential measurement was performed at 25°C on a Malvern Zetasizer-Nano Z instrument. The cellular bioimaging was conducted by the Leica Stellaris 5 confocal laser scanning microscope. The bioimaging for organs and animals were performed with an IVIS Spectrum (PerkinElmer) in fluorescence mode equipped with 675-nm filters for excitation and 700 to 720 and 760 to 780 nm for emission, respectively. Quantitative measurements of the fluorescence signal were processed with Living Image V.4.5. BioRender supports the schematic diagram of mice and organs in this paper.

### Synthesis of RP-SC

Hcy-BOH (2.0 mg) and Cy-Dopa (2.2 mg) was dissolved in 500 μl of dimethyl sulfoxide (DMSO) and added to 40 mg of l-serine LMWC dissolved in 5.0 ml of deionized water. After stirring at room temperature for 24 hours in the dark, the mixture was dialyzed [molecular weight cut-off (MWCO) 2000] for 48 hours at room temperature in the dark, followed by lyophilization. A 5.0 mg of freeze-dried mixture was dissolved in 5.0 ml of 0.5% acetic acid solution, and 2.0 ml of sodium tripolyphosphate solution (TPP, 1.25 mg/ml) was added dropwise. After stirring at 1000 rpm, the conjugation reaction was maintained for 30 min and then purified by successive dialysis (MWCO 5000) against deionized water for 48 hours. Then, the product solution was adjusted to 7.4 and freeze-dried. The amounts of Hcy-BOH and Cy-Dopa conjugated on RP-SC were determined by UV-Vis spectrophotometry and analytical HPLC. The encapsulation efficiency and loading content of RP-SC were determined according to a reported method (*23*). The detailed synthesis procedures can be obtained in the Supplementary Materials.

### Absorption and fluorescence analysis

Probes were dissolved in anhydrous DMSO to obtain 1.0 mM stock solutions. These stock solutions were diluted with degassed 0.10 M sodium phosphate buffer (PBS, pH = 7.4) to a final concentration. H_2_O_2_ was diluted from 30% solution, and the concentration was determined from absorption at λ = 240 nm (ε = 43.6 M^−1^ cm^−1^).

Absorption and fluorescence spectra were obtained with 1.0-cm quartz cells. The excitation and emission slits were both set at 15 nm. The fluorescence intensity was measured at 706 and 766 nm for Hcy-BOH and Cy-Dopa, respectively. Relative fluorescence quantum efficiency was obtained by comparing the area under the emission spectrum of the test sample with that of a solution of Cy5.5 in PBS (Φ = 0.23) and Cy7 in PBS (Φ = 0.01).

### Cells incubation and imaging information

Human proximal tubular cells (HK-2) were cultured in RPMI 1640 supplemented with 10% fetal bovine serum and 1% penicillin-streptomycin. All cells were cultured at 37°C in a CO_2_/air (5%/95%) incubator. After grown to 90% confluent, cells were passed in a ratio of 1:2. The cells were incubated with RP-SC (100 μg/ml) for 4 hours before washing thrice with PBS for confocal experiments.

### MTT assay for the evaluation of cytotoxicity

The cytotoxicity of probes was analyzed by MTT assays. HK-2 cells were seeded onto the 96-well plate and incubated in DMEM containing probe RP-SC of various concentrations (0, 0.1, 0.2, 0.5, 1.0, 2.0, and 4.0 mg/ml) at 37°C for 24 hours. HK-2 cells were incubated with MTT solution (5.0 mg/ml) for 4 hours in a CO_2_/air (5%/95%) incubator. A 80 μl of DMSO was added to each well to dissolve the formazan before analyzing the absorbance of each well at the wavelength of 565 nm with a microplate reader.

### KIM-1 targeting ability

HK-2 cells were seeded into 12-well plates and incubated overnight. After stimulation with cisplatin (50 μM) for 24 hours, HK-2 cells were treated with a blocking antibody to KIM-1 at a concentration of 30 μg/ml for 1 h and then administrated with RP-SC or RP-C at a concentration of 100 μg/ml for another 4 hours. HK-2 cells expressing KIM-1 were treated with KIM-1 antibody (1:100; GeneTex, GTX85068) and then to FITC-labeled secondary antibodies. The staining was examined using confocal laser scanning microscope (Leica STELLARIS 5). Cisplatin-stimulated cells without blocking treatment of KIM-1 antibody were also tested as control.

### Western blot

HK-2 cells were lysed according to the lysis protocol in ice-cold radioimmunoprecipitation assay (*34*). The concentrations of proteins were tested using a bicinchoninic acid protein assay kit. Equal amounts of proteins for each group were loaded on SDS–polyacrylamide gel electrophoresis gels (8%). Following electrophoresis, the membranes were then probed overnight at 4°C with primary antibodies for KIM-1 (1:1000; GeneTex) and β-actin (1:50,000; GeneTex). After being washed, the membrane was incubated with appropriate secondary antibody [1:5000; the secondary anti-rabbit immunoglobulin G (IgG) horseradish peroxidase] for 1 hour at room temperature. The secondary antibody was imaged using the chemical illuminant (Omni-ECL, epizyme) and quantified in a Bio-Rad system (ChemiDoc Touch Imaging System).

### AKI model construction

All animal experiments were approved by the Institutional Animal Ethics Committee at Shanghai Jiao Tong University (approval number: A2024104). All animal handling procedures were performed strictly in accordance with the P.R. China Legislation on the Use and Care of Laboratory Animals. Female BALB/c nude mice (6 to 8 weeks old) were purchased from Shanghai SLAC Laboratory Animal Co. Ltd.

After an adaptation period of 1 week, BALB/c female nude mice (20 ± 2.0 g) were randomly divided into different groups (five mice in each group). Mice receiving intraperitoneally injection of saline (100 μl) were chose as the control group. Mice were treated with cisplatin (20 mg kg^−1^ body weight, intraperitoneally injection) for 48 hours to establish mice models with AKI. For the NAC treatment group, AKI mice were intraperitoneally injected with NAC at a dose of 100 mg/kg. AKI mice were injected with NAC for 1, 2, and 3 days corresponding to 1×, 2×, and 3× groups, respectively. All mice have fasted for 20 hours before orbital blood collection. Last, all animals were euthanized by cervical dislocation, and kidneys were taken for imaging and histopathological analysis. Serum biochemical indices (BUN and SCr) were detected using BUN (Solarbio, BC1530) and SCr (Yuanye, R21727) kits according to the product instructions.

### Pharmacokinetic studies

Normal mice were intravenously injected with 0.1 ml of RP-SC dissolved in PBS buffer, and blood sample (200 μl) was collected from tail vein at 0, 0.5, 1, 2, 4, 8, and 12 hours postinjection. Blood was collected in heparinized capillary tubes and centrifuged at 3500 rpm for 10 min. After removing the protein, the amount of RP-SC in blood serum was quantified using analytical HPLC, and the relative amount was plotted against postinjection time. The points were then fitted by a biexponential decay curve to estimate elimination half-life (*t*_1/2_).

### Hemolytic studies

Fresh mice blood was diluted by saline, and the red blood cells were isolated from serum by centrifugation. The red blood cells were further washed with saline for three times, before serial concentrations of RP-SC (0.05, 0.1, 0.2, 0.5, 1.0, 2.0, and 4.0 mg/kg) was added into the red blood cells suspension. PBS and Triton X-100 (a common surfactant for cell lysis, 10 g/liter) were used as negative or positive controls, respectively. The solution was mixed by vortex and then incubated at 37°C for 2 hour. Afterward, red blood cells were centrifuged at 3000 rpm for 10 min, and the supernatant of each sample was transferred into a 96-well plate. Free hemoglobin in the supernatant was measured with a Microplate reader at 540 nm. The hemolysis ratio of red blood cells was calculated using the following formula: hemolysis (%) = (*A*_sample_ − *A*_negative control_)/(*A*_positive control_ − *A*_negative control_).

### In vivo biodistribution and renal clearance efficiency studies

Mice were intravenously injected with 0.1 ml of RP-SC dissolved in PBS buffer and immediately placed in metabolic cages. Urine was collected at 2, 4, 6, 12, and 24 hours postinjection, centrifuged at 4500 rpm for 10 min, and filtered by a 0.22-μm syringe filter. After collecting urine 24 hours after RP-SC injection, the mice were humanely euthanized, and major organs (kidney, heart, lung, liver, spleen, intestine and brain) were collected. The collected organs were homogenized in PBS buffer and centrifuged at 4500 rpm for 15 min to remove insoluble components. Quantifications of RP-SC in the urine excretion and homogenate of major organs were done using analytical HPLC (*41*).

### Histopathology

All tissues were fixed in 4% paraformaldehyde for 24 hours, dehydrated in ethanol with increasing concentrations, cleared with xylene, and last embedded in paraffin. The tissue-embedded wax was cut into 5-μm sections and stained with H&E. The stained tissue sections were microscopically determined to identify areas of tissue injury. For immunofluorescence staining, the sections were incubated with KIM-1 antibody (1:100; Abcam, ab316854) for 12 hours at 4°C. After being washed with PBS to remove unbound antibody, the sections were counterstained with FITC-labeled Goat Anti-Rabbit IgG (H+L) (1:500; Beyotime) for 60 min at room temperature. Next, the cell nuclei were stained with 4′,6-diamidino-2-phenylindole. All the stained sections were imaged using an Olympus VS200 Research Slide Scanner.

## Supplementary Materials

### This PDF file includes:

Figs. S1 to S29 Supplementary Spectra
