# Supplementary material for: Activatable fluorescent ratiometric probes for early diagnosis and prognostic assessment of acute kidney injury
Source: Sci Adv. 2025 Oct 22;11(43):eaea1654. doi: 10.1126/sciadv.aea1654 (PMC12542964; doi:10.1126/sciadv.aea1654)
Supplement: Supplementary file 1 — Figs. S1 to S29 Supplementary Spectra [file sciadv.aea1654_sm.pdf]

Science Advances
AAAS

Supplementary Materials for

Activatable fluorescent ratiometric probes for early diagnosis and prognostic assessment of acute kidney injury

Ni Li *et al.*

Corresponding author: Dalong Ni, ndl12353@rjh.com.cn

Sci. Adv. **11**, eaea1654 (2025)
DOI: 10.1126/sciadv.aea1654

This PDF file includes:

Figs. S1 to S29
Supplementary Spectra

1. Supporting Figures

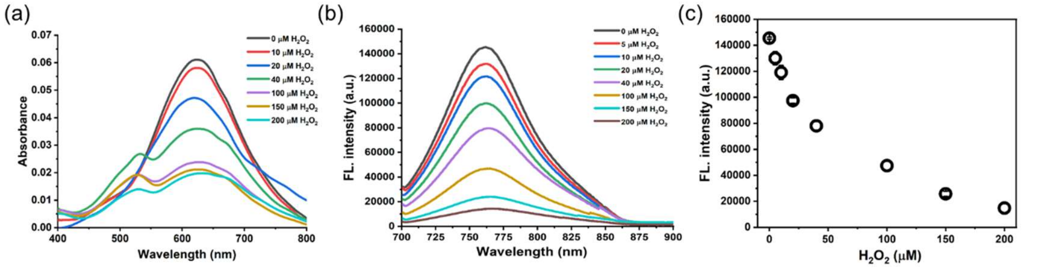

Figure S1. Physicochemical properties of Cy-Dopa. (a) Absorption, (b) fluorescence spectra, and (c) dose-response of Cy-Dopa (10.0 μM) upon addition of H_2O_2. Solvent: 0.10 M PBS, pH = 7.4, containing 0.2% DMSO as a co-solvent.

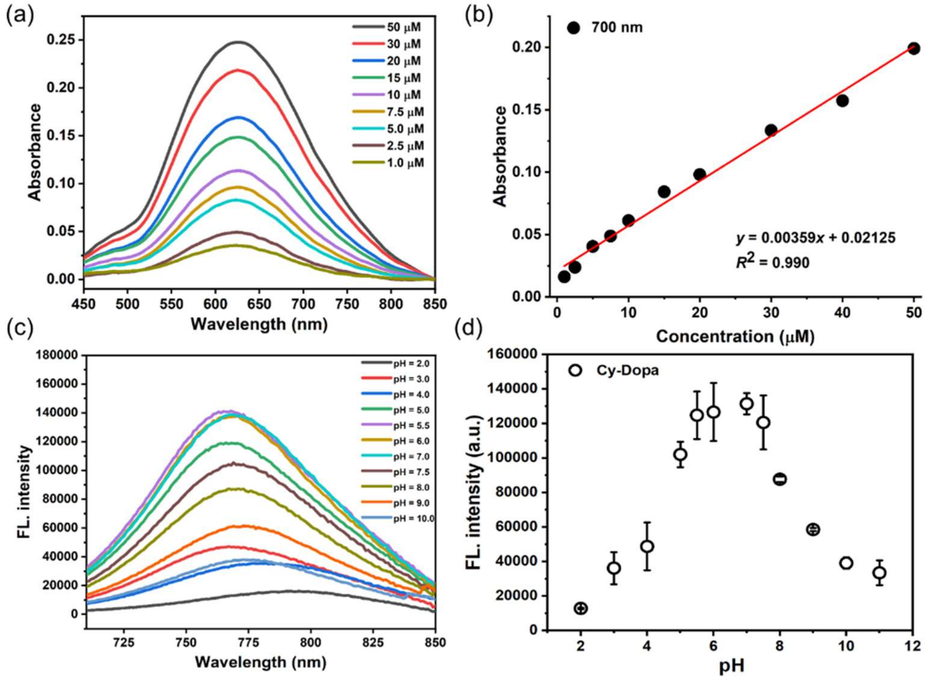

Figure S2. Solubility and pH stability tests of Cy-Dopa. (a) The absorption spectra of different concentrations of probe Cy-Dopa. (b) Absorbance at 700 nm vs. the concentrations of probe Cy-Dopa (0 - 50 μM) in PBS solution (0.10 M, pH = 7.4). (c) The fluorescence spectrum of the probe Cy-Dopa in different pH solutions and (d) the fluorescence intensity at 766 nm in different pH solutions.

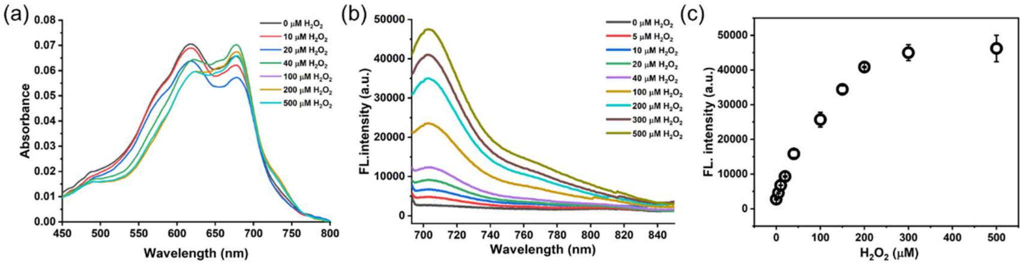

Figure S3. Physicochemical properties of Hcy-BOH. (a) Absorption, (b) fluorescence spectra, and (c) dose-response of Hcy-BOH (5.0 μM) upon addition of H_2O_2. Solvent: 0.10 M PBS, pH = 7.4, containing 0.2% DMSO as a co-solvent.

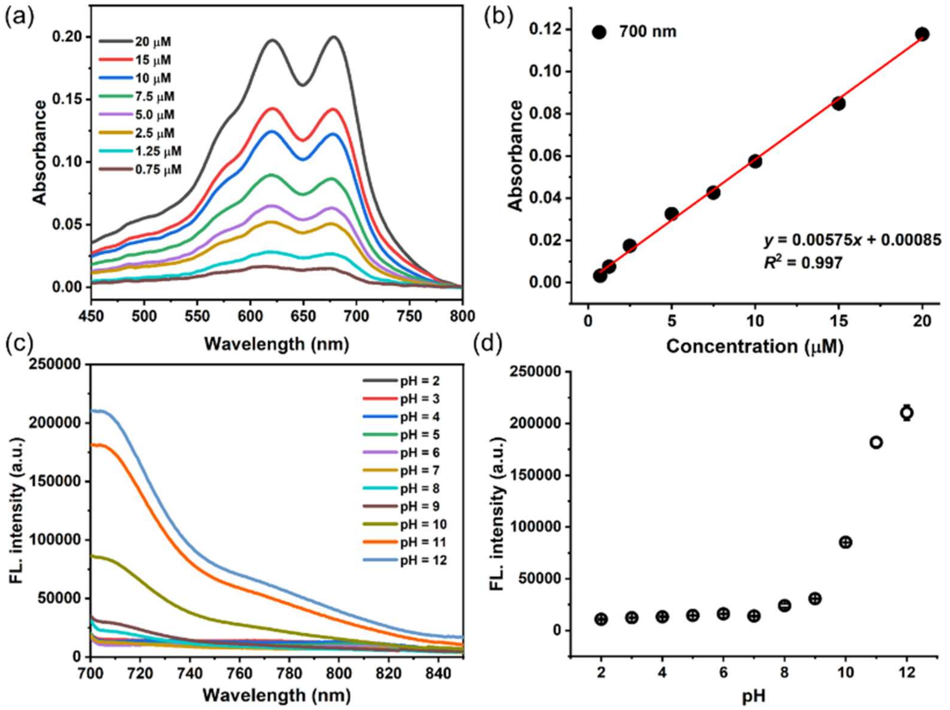

Figure S4. Solubility and pH stability tests of Hcy-BOH. (a) The absorption spectra of different concentrations of probe Hcy-BOH. (b) Absorbance at 700 nm vs. the concentrations of probe Hcy-BOH (0 - 20 μM) in PBS solution (0.10 M, pH = 7.4). (c) The fluorescence spectrum of the probe Hcy-BOH in different pH solutions and (d) the fluorescence intensity at 706 nm in different pH solutions.

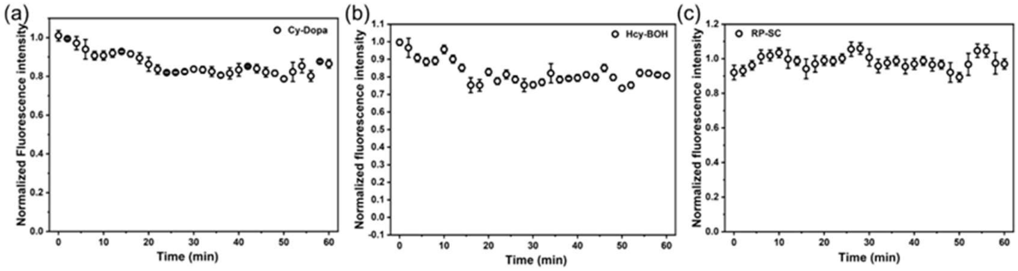

Figure S5. Photostability tests of Cy-Dopa, Hcy-BOH and RP-SC. Normalized fluorescence intensity of (a) Cy-Dopa and (b) Hcy-BOH (5.0 μM in 0.20 M PBS, pH = 7.4) and (c) RP-SC (20 μg/mL in 0.20 M PBS, pH = 7.4) over time upon irradiation.

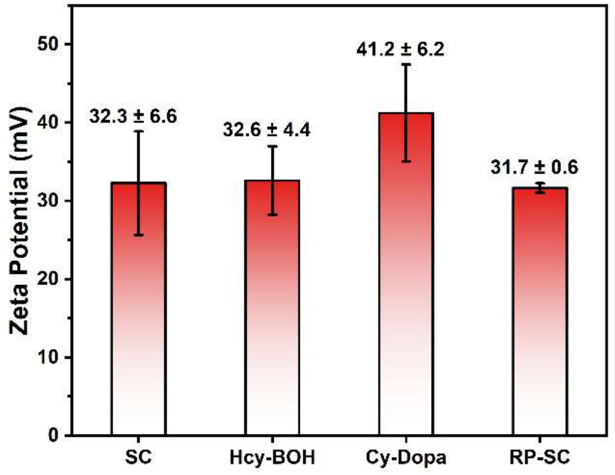

Figure S6. The zeta potentials of *L*-serine-LMWC (SC), Hcy-BOH, Cy-Dopa, and RP-SC.

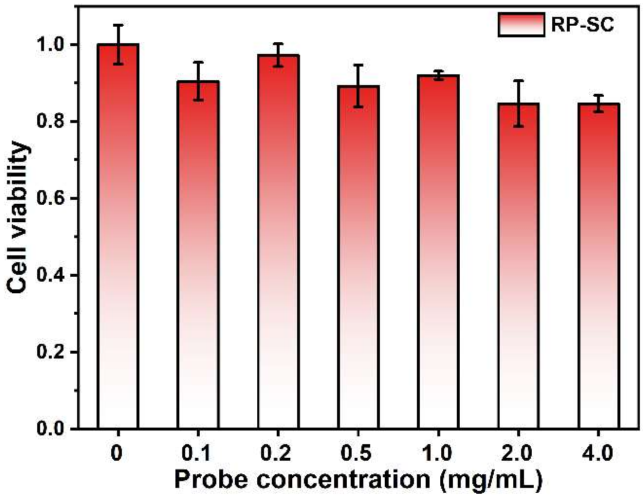

Figure S7. Cytotoxicity test of RP-SC. Cell viability assay of HK-2 cells after being incubated with different concentrations of RP-SC at 37 °C for 24 h.

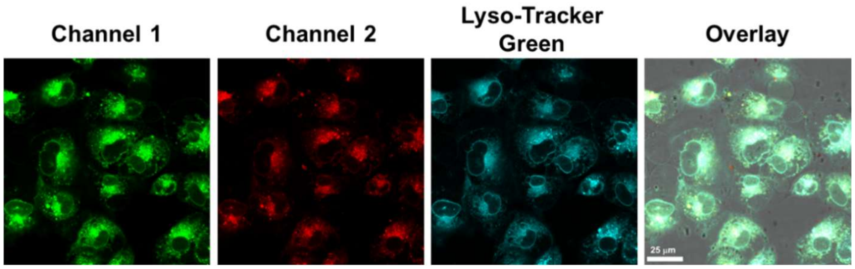

Figure S8. Co-staining imaging of RP-SC. Confocal fluorescence images of HK-2 cells co-stained with RP-SC (100 μg/mL) and Lyso-Tracker Green (500 nM). The Pearson's correlation R_r was 0.82. Scale bar: 25 μm.

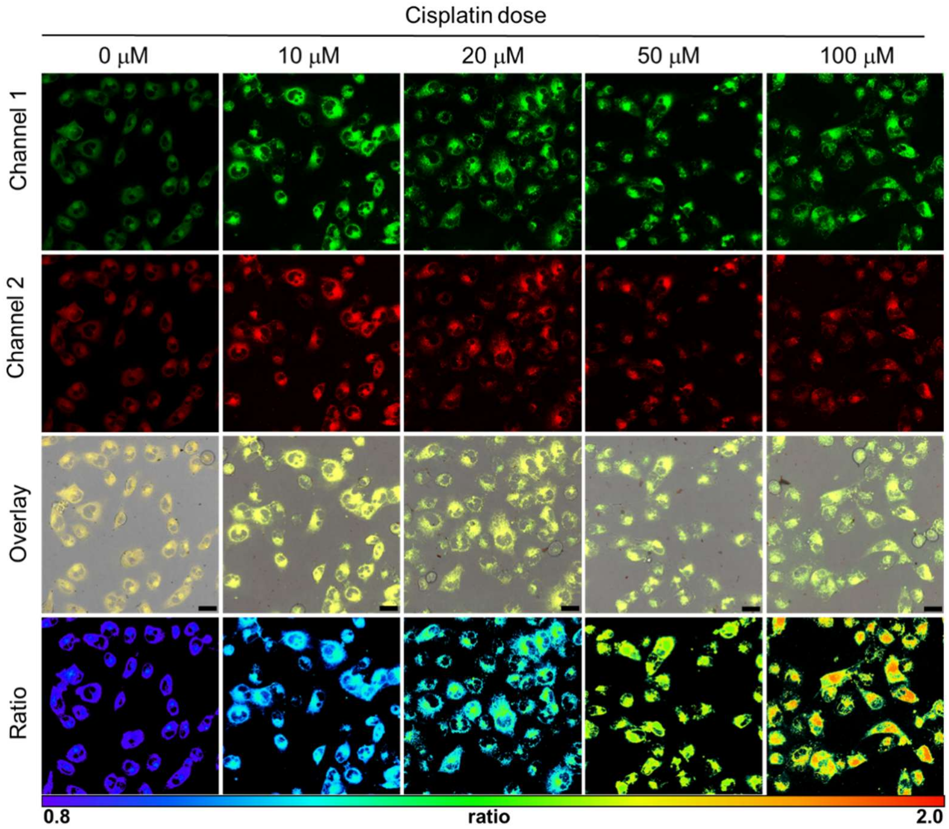

Figure S9. Detection of H_2O_2 in HK-2 cells induced with cisplatin by RP-SC. Ratiometric fluorescence images of living HK-2 cells treated with different doses of cisplatin (0-100 μM). Scale bar: 25 μm.

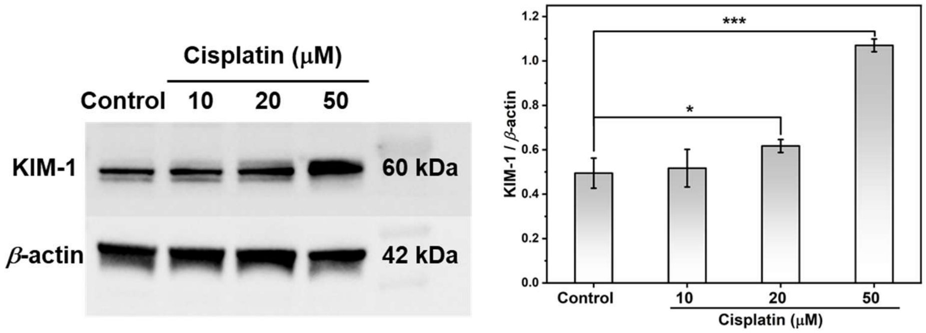

Figure S10. KIM-1 protein expression in HK-2 cells treated with cisplatin. Western blotting analysis of KIM-1 expression using lysates from normal and cisplatin-stimulated HK-2 cells.

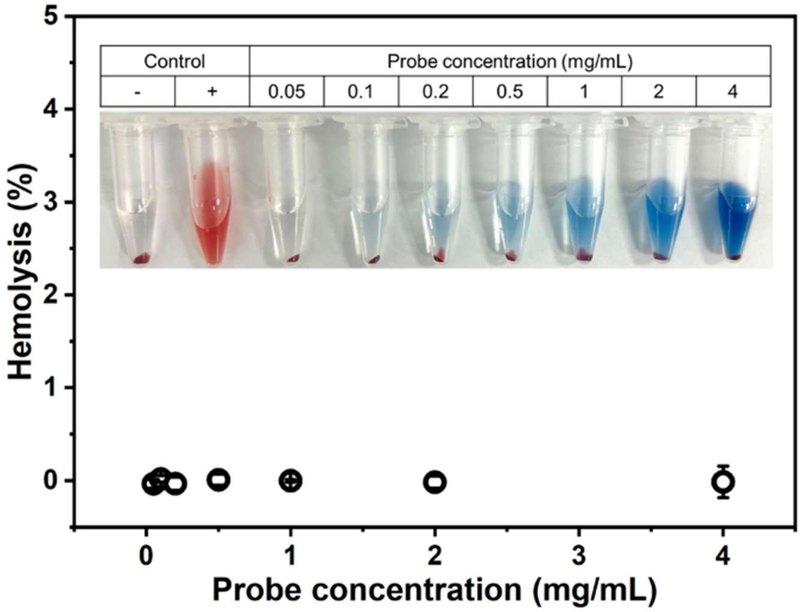

Figure S11. Hemolysis test of RP-SC in fresh mice blood.

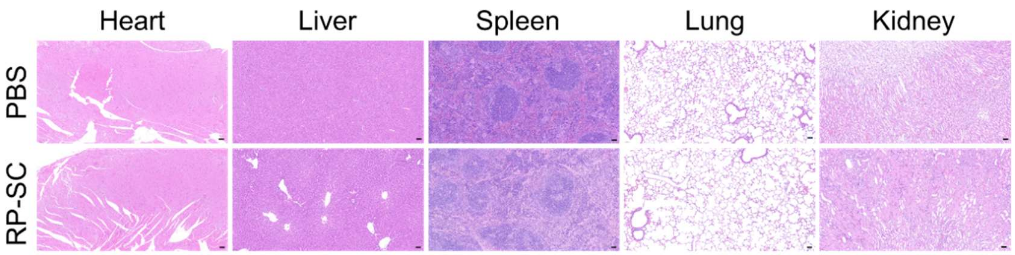

Figure S12. Tissue toxicity test of RP-SC. H&E staining of main organs resected from healthy mice after intravenous injection of **RP-SC** (1.0 mg/mL, 100 μL) or PBS for 24 h. Scale bar: 50 μm.

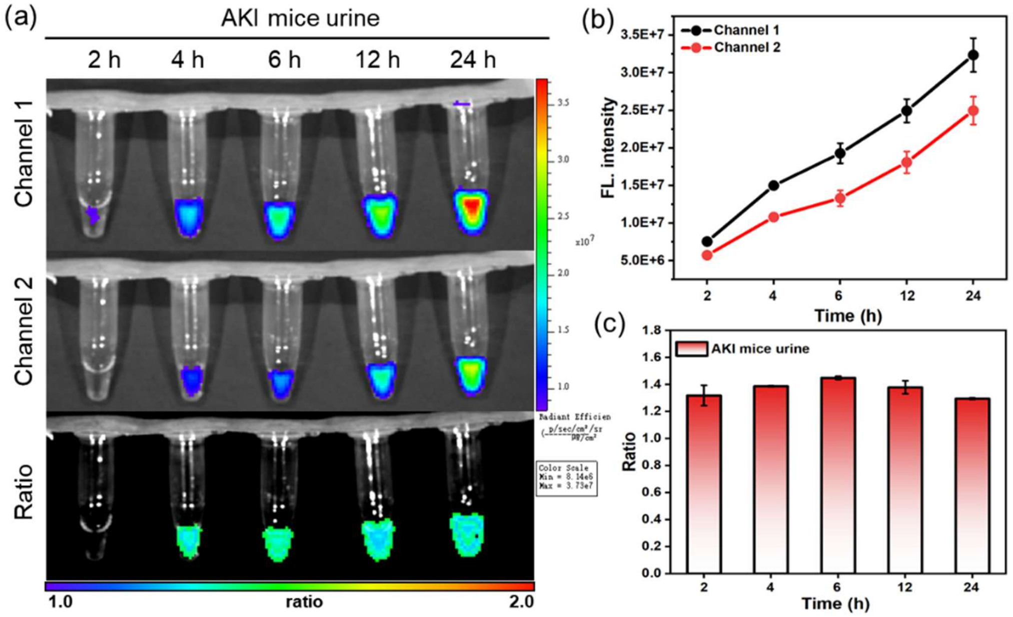

Figure S13. Urinalysis of RP-SC in AKI mice. (a) Ratiometric fluorescence images of AKI mice urine over time after intravenous injection of RP-SC probe (1.0 mg/mL, 100 μL). Fluorescence intensity in channel 1 and channel 2 (b) and ratio (c) of AKI mice urine over time after intravenous injection of RP-SC probe.

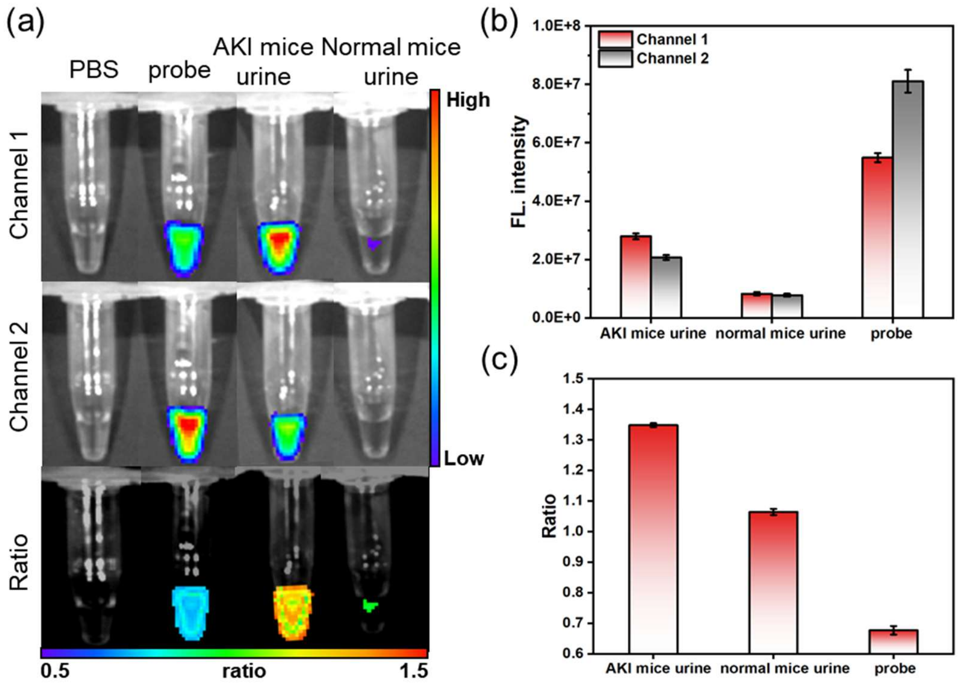

Figure S14. Urinalysis of RP-SC in AKI mice and normal mice. (a) Ratiometric fluorescence images of PBS, probe RP-SC, AKI mice urine and normal mice urine after intravenous injection of RP-SC probe (1.0 mg/mL, 100 μL) for 24 h. Fluorescence intensity (b) and ratio (c) of PBS, RP-SC probe, AKI mice urine and normal mice urine.

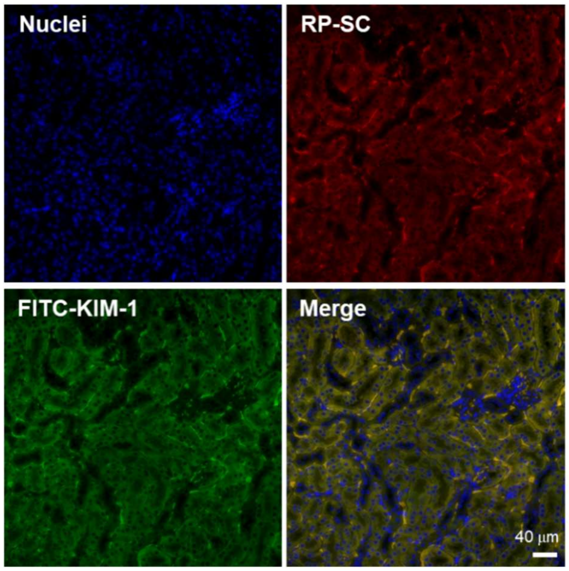

Figure S15. Co-localization imaging of KIM-1 and RP-SC. Representative confocal images of kidney sections from AKI mice after intravenous injection of RP-SC (red signal) for 4 h. Blue indicated DAPI staining. Immunostaining for Kim-1 was shown in green. Scale bar: 40 μm.

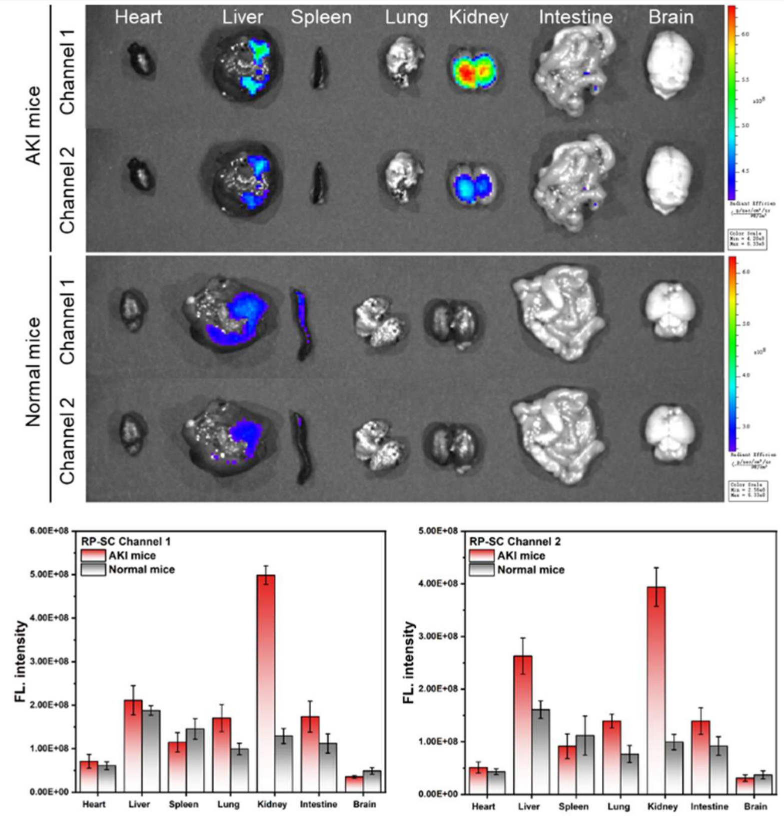

Figure S16. Organ imaging of RP-SC. Fluorescence images and intensity of main organs (heart, liver, spleen, lung, kidney, intestine and brain) after intravenous injection of RP-SC (1.0 mg/mL, 100 μL) for 12 h.

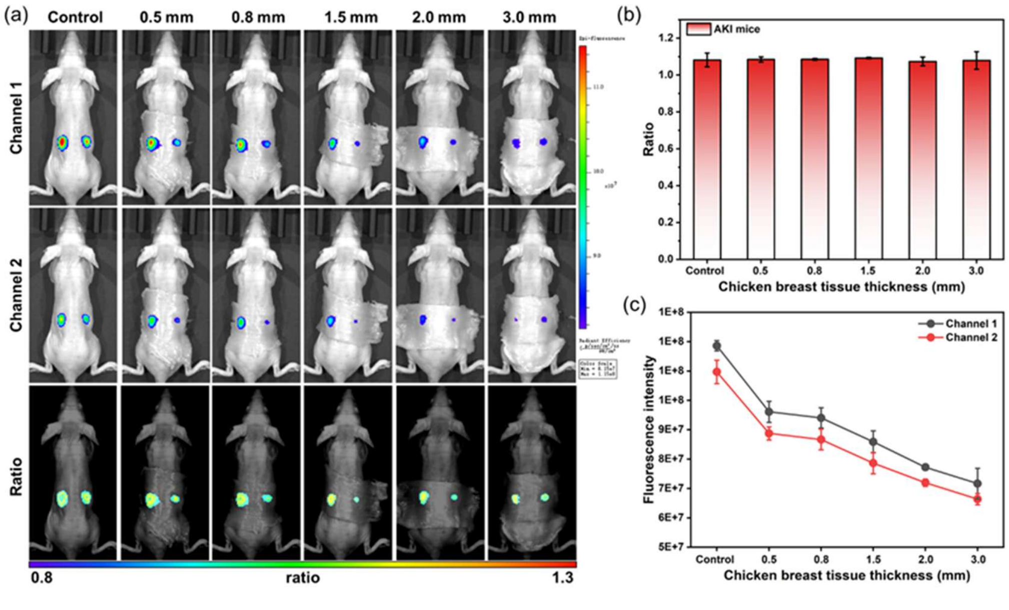

Figure S17. Photopenetration of RP-SC. (a) Ratiometric fluorescence images, (b) ratio and (c) $F_{\text{channel 1}}$ & $F_{\text{channel 2}}$ of renal areas in the AKI mice covered by chicken breast tissue with different thicknesses. λ_{ex} = 675 nm; channel 1: λ_{em} = 700~720 nm; channel 2: λ_{em} = 760~780 nm.

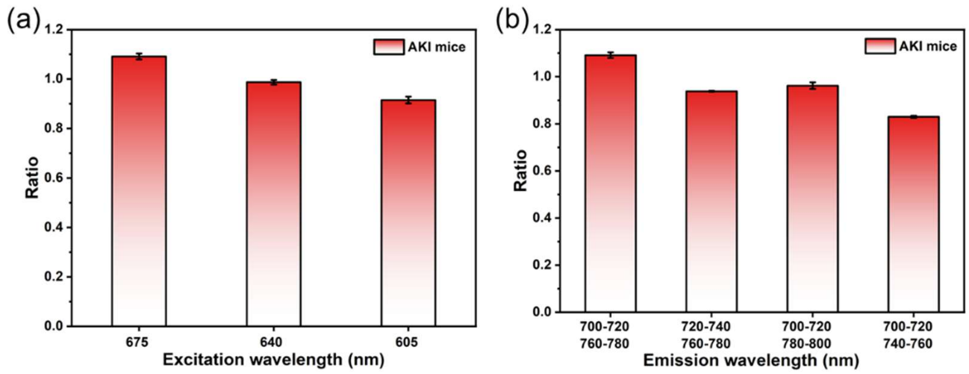

Figure S18. The renal fluorescence ratio of AKI mice under different wavelengths of excitation and emission.

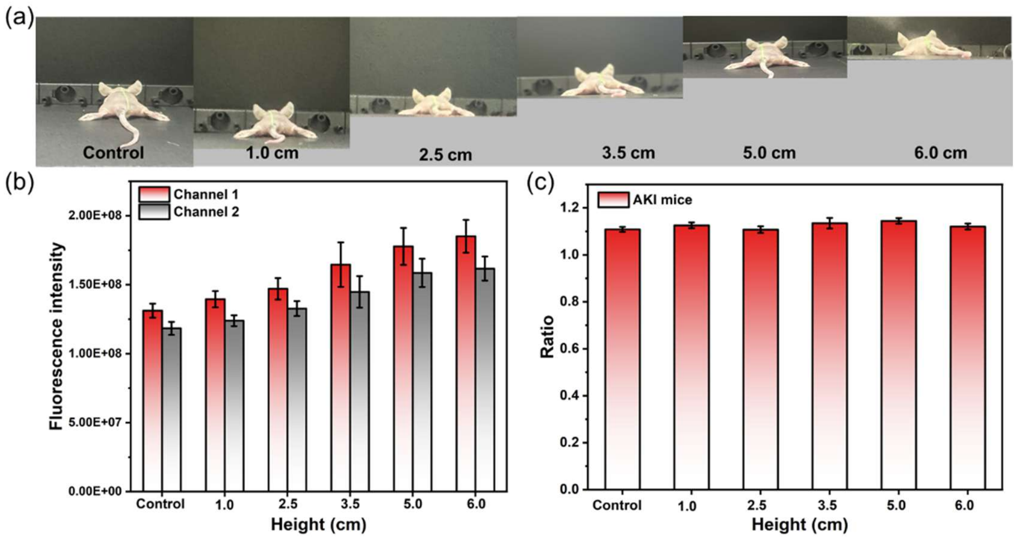

Figure S19. Imaging at different distances between the excitation light and the mice. (a) Set-ups for ratiometric fluorescence imaging, (b) $F_{channel\ 1}$ & $F_{channel\ 2}$, and (c) the renal fluorescence ratio of AKI mice under excitation at different distances. λ_{ex} = 675 nm; channel 1: λ_{em} = 700~720 nm; channel 2: λ_{em} = 760~780 nm.

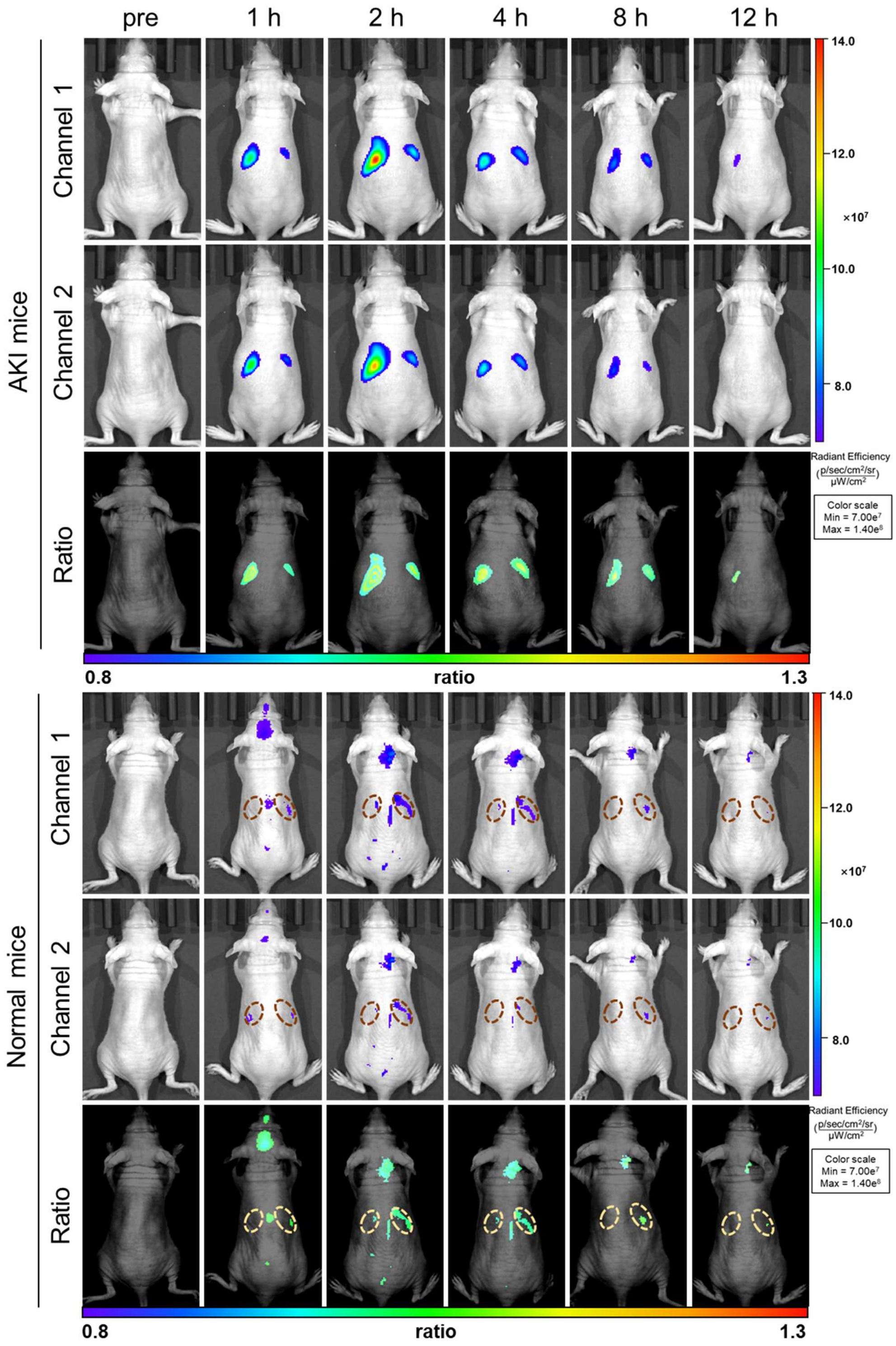

Figure S20. *In vivo* imaging of RP-C. Ratiometric fluorescence images of AKI mice or normal mice prior (pre) or at 1, 2, 4, 8, and 12 h post intravenous injection of RP-C (1.0 mg/mL, 100 μL).

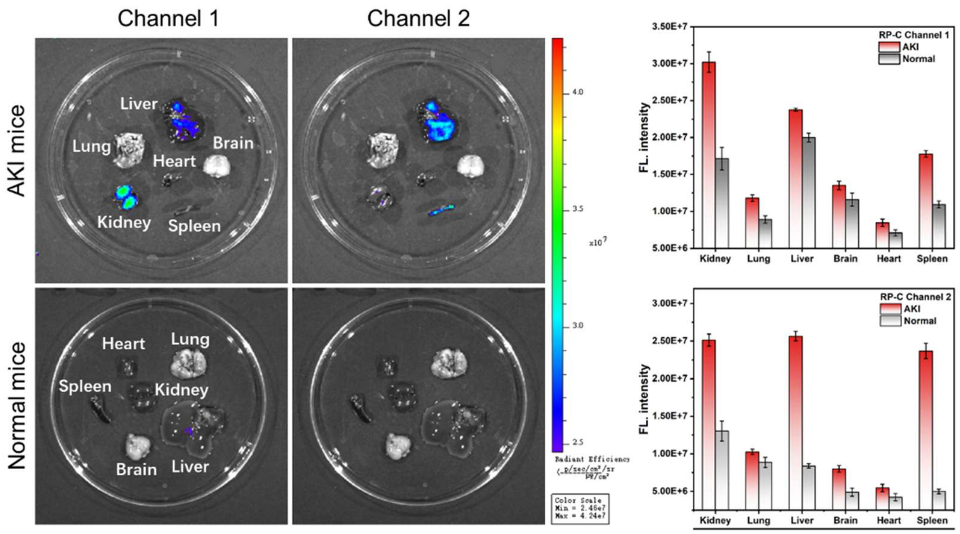

Figure S21. Organ imaging of RP-C. Fluorescence images and intensity of main organs (heart, liver, spleen, lung, kidney and brain) after intravenous injection of RP-C (1.0 mg/mL, 100 μL) for 12 h.

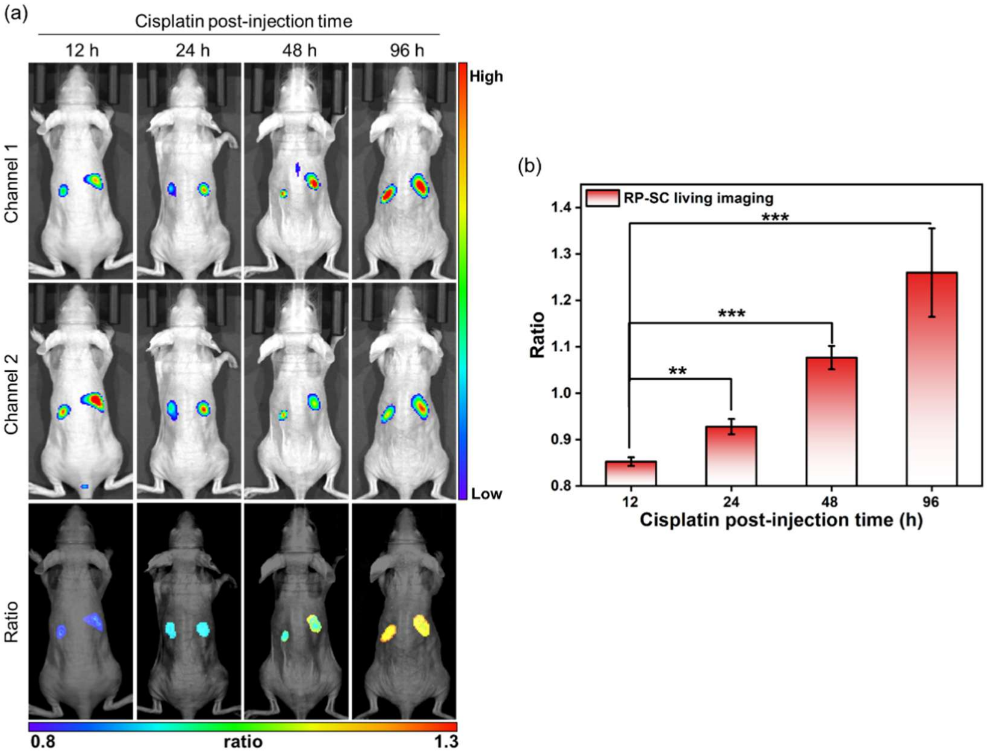

Figure S22. Monitoring of renal function in AKI mice after different durations of cisplatin treatment. Ratiometric fluorescence imaging (a) and ratio (b) of mice receiving intraperitoneal injection of cisplatin (20 mg/kg) at 12, 24, 48, and 96 h, followed by intravenous injection of RP-SC (1.0 mg/mL, 100 μL) at 4 h.

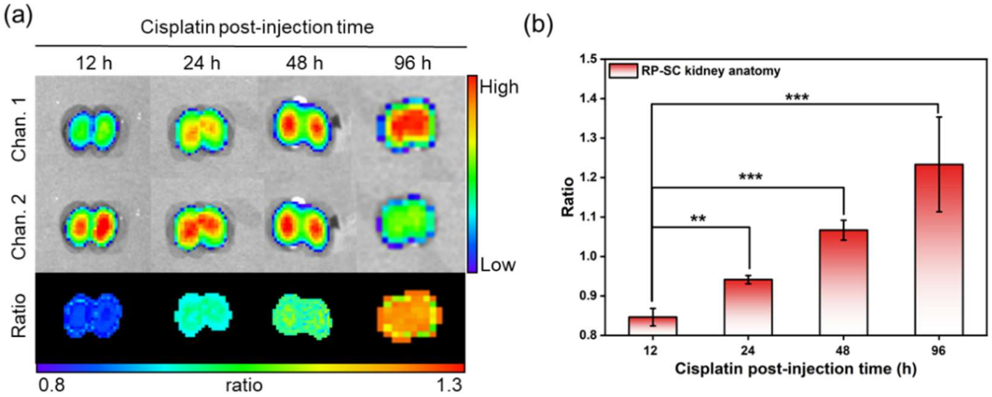

Figure S23. Renal imaging of AKI mice after different durations of cisplatin treatment. Ratiometric fluorescence imaging (a) and ratio (b) of anatomical kidneys receiving intraperitoneal injection of cisplatin (20 mg/kg) at 12, 24, 48, and 96 h, followed by intravenous injection of RP-SC (1.0 mg/mL, 100 μL) at 4 h.

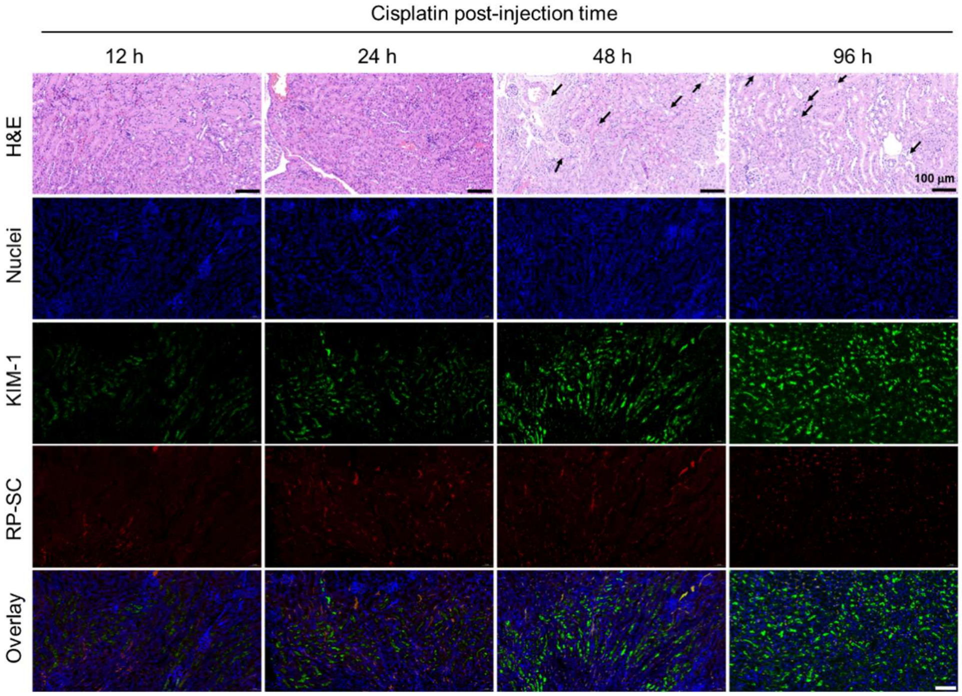

Figure S24. Renal slices analysis of AKI mice after different durations of cisplatin treatment. H&E staining and immunofluorescence staining of kidney tissue slices resected from mice at 12, 24, 48, and 96 h post-treatment with cisplatin (20 mg/kg). Scale bar: 100 μm.

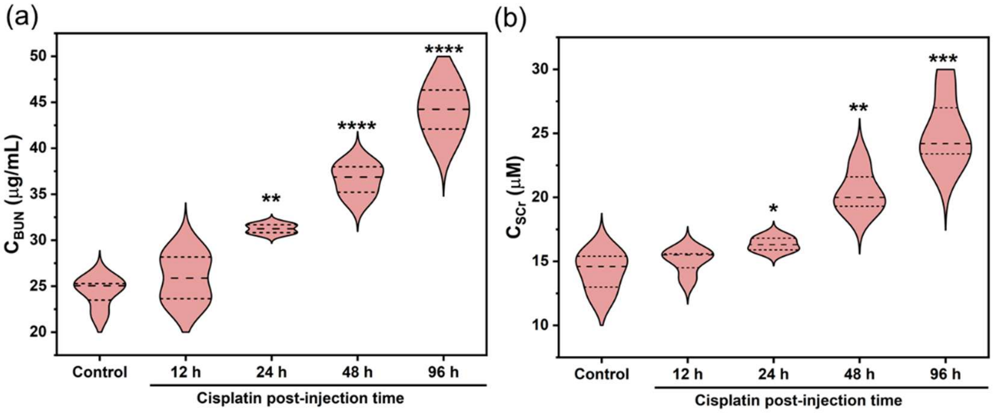

Figure S25. Blood biomarkers of AKI mice after different durations of cisplatin treatment. The concentrations of (a) BUN and (b) SCr in serum from AKI mice receiving intraperitoneal injection of cisplatin (20 mg/kg) at 12, 24, 48, and 96 h.

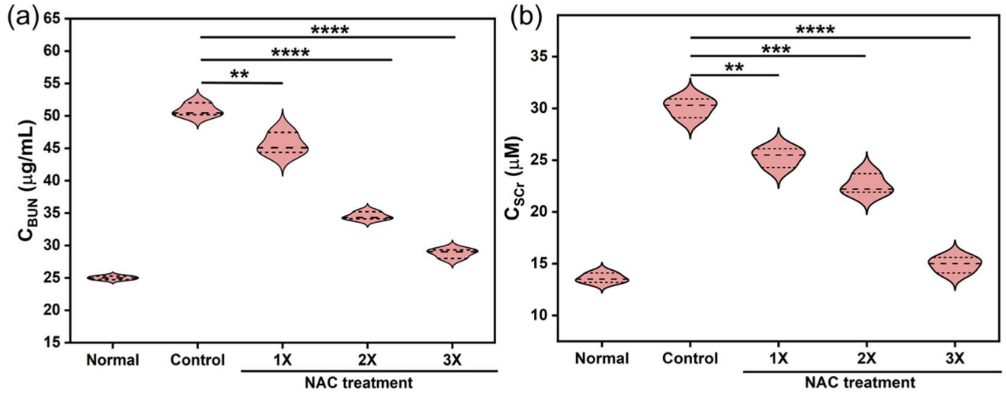

Figure S26. Blood biomarkers of AKI mice after different doses of NAC treatment. The concentrations of (a) BUN and (b) SCr in serum from AKI mice receiving different doses of NAC treatment.

2. Synthesis

NMR abbreviations:

s = singlet

d = doublet

dd = doublet of doublets

t = triplet

dt = doublet of triplets

td = triplet of doublets

q = quartet

p = quintuplet

m = multiplet

Reagent abbreviations:

EtOAc = ethyl acetate

DMF = *N*, *N*-dimethylformamide

DCM = dichloromethane

MeCN = acetonitrile

TFA = trifluoroacetic acid

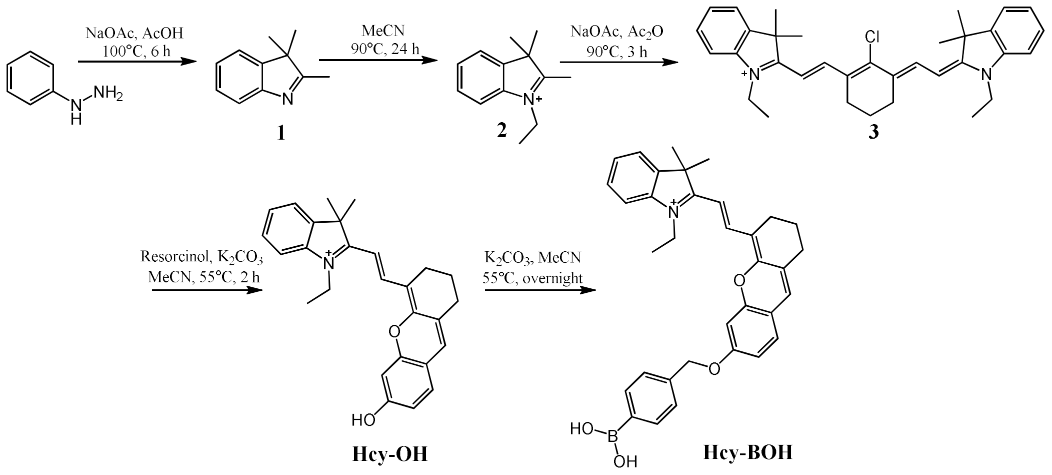

Figure S27. Synthesis of Hcy-BOH.

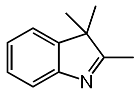

1

To a dried flask, phenylhydrazine (5.41 g, 50 mmol), 3-methyl-butan-2-one (4.74 g, 55 mmol), NaOAc (4. 92 g, 60 mmol), and acetic acid (30.0 mL) were added. The mixture solution was refluxed at 100 °C for 6 h. After completion of the reaction, the mixture was extracted with EtOAc. The combined organic layer was washed with saturated aqueous $NaHCO_3$ (3 × 10.0 mL) and brine (3 × 10.0 mL), and dried over anhydrous Na_2SO_4. After solvent evaporation under reduced pressure, the crude product was purified by column chromatography to provide **compound 1** (5.00 g, 63%).

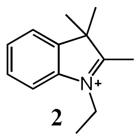

To a dried flask, compound 1 (3.18 g, 20 mmol), iodoethane (3.43 g, 22 mmol) and MeCN (30.0 mL) were added. The mixture solution was refluxed at 90 °C for 24 h. After completion of the reaction, the mixture was extracted with EtOAc. The combined organic layer was washed with brine (3 × 10.0 mL), and dried over anhydrous Na_2SO_4. After solvent evaporation under reduced pressure, the crude product was purified by column chromatography to provide **compound 2** (2.68 g, 71%). ^{1}H NMR (400 MHz, Chloroform-*d*) δ 7.72 (q, *J* = 4.1 Hz, 1H), 7.55 (q, *J* = 5.5, 4.8 Hz, 3H), 4.70 (q, *J* = 7.4 Hz, 2H), 3.11 (s, 3H), 1.60 (m, 9H). ^{13}C NMR (101 MHz, Chloroform-*d*) δ 195.43, 141.71, 140.58, 130.19, 129.60, 123.46, 115.39, 54.68, 45.48, 23.13, 17.03, 13.65.

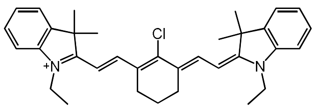

3

To a dried flask, compound 2 (526 mg, 2.80 mmol), (*E*)-2-Chloro-3-(hydroxymethylene)cyclohex-1-ene-1-carbaldehyde (266 mg, 1.54 mmol), NaOAc (689 mg, 8.40 mmol) and Ac_2O (10.0 mL) were added. The mixture solution was stirred at 90 °C for 3 h. After completion of the reaction, the mixture was extracted with EtOAc. The combined organic layer was washed with brine (3 × 10.0 mL), and dried over anhydrous Na_2SO_4. After solvent evaporation under reduced pressure, the crude product was purified by column chromatography to provide **compound 3** (320 mg, 42%). ^{1}H NMR (400 MHz, Chloroform-*d*) δ 8.37 (d, *J* = 14.1 Hz, 2H), 7.40 (d, *J* = 7.4 Hz, 4H), 7.30 – 7.25 (m, 2H), 7.21 (d, *J* = 7.7 Hz, 2H), 6.20 (d, *J* = 14.1 Hz, 2H), 4.25 (q, *J* = 7.2 Hz, 4H), 2.75 (t, *J* = 6.2 Hz, 4H), 2.11 (s, 2H), 1.73 (s, 12H), 1.47 (t, *J* = 7.2 Hz, 6H). ^{13}C NMR (101 MHz, Chloroform-*d*) δ 171.94, 150.67, 144.53, 141.73, 141.14, 128.90, 127.34, 125.39, 122.34, 110.77, 100.93, 49.38, 40.07, 28.07, 26.74, 20.96, 20.69, 12.48.

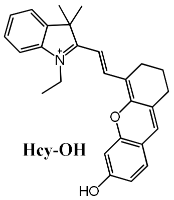

Hcy-OH

K_2CO_3 (243 mg, 1.76 mmol) and resorcinol (130 mg, 1.18 mmol) were reacted in MeCN for 30 min. The MeCN solution of compound 3 (300 mg, 0.59 mmol) was added to the above solution in a dropwise fashion, and the reaction mixture was stirred for 2.0 h at 55 °C. After completion of the reaction, the mixture was extracted with DCM. The combined organic layer was washed with brine (3 × 10.0 mL), and dried over anhydrous Na_2SO_4. After solvent evaporation under reduced pressure, the crude product was purified by column chromatography to provide **compound Hcy-OH** (105 mg, 45%). ^{1}H NMR (400 MHz, Methanol-*d*$_4$) δ 8.48 (d, *J* = 14.2 Hz, 1H), 7.52 (d, *J* = 6.8 Hz, 1H), 7.46 – 7.38 (m, 2H), 7.36 – 7.24 (m, 3H), 6.74 (dd, *J* = 8.7, 2.2 Hz, 1H), 6.62 (d, *J* = 2.1 Hz, 1H), 6.16 (d, *J* = 14.2 Hz, 1H), 4.18 (q, *J* = 7.1 Hz, 2H), 2.68 (dt, *J* = 23.1, 6.1 Hz, 4H), 1.89 (p, *J* = 6.3 Hz, 2H), 1.73 (s, 6H), 1.40 (t, *J* = 7.2 Hz, 3H). ^{13}C NMR (101 MHz, Methanol-*d*$_4$) δ 172.64, 171.07, 162.35, 156.62, 141.78, 141.25, 141.08, 138.13, 129.46, 128.51, 124.99, 122.59, 122.15, 119.29, 114.57, 114.51, 110.48, 102.04, 98.93, 49.21, 38.88, 28.17, 27.27, 23.81, 20.57, 11.01.

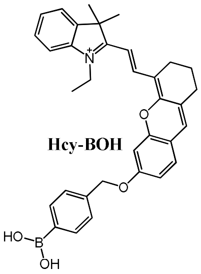

Hcy-BOH

In a dry reaction flask, compound Hcy-OH (105 mg, 0.26 mmol), K_2CO_3 (73 mg, 0.53 mmol), and anhydrous MeCN (10.0 mL) as the solvent were added successively. Then the MeCN solution of *4*-(Bromomethyl)phenylboronic acid (114 mg, 0.53 mmol) was slowly added in a dropwise fashion to the reaction solution at 0 °C. The reaction mixture was stirred overnight at 55 °C. After completion of the reaction, the mixture was extracted with DCM. The combined organic layer was washed with brine (3 × 10.0 mL), and dried over anhydrous Na_2SO_4. After solvent evaporation under reduced pressure, the crude product was purified by column chromatography to provide **compound Hcy-BOH** (82 mg, 59%). ^{1}H NMR (400 MHz, Methanol-*d*$_4$) δ 8.70 (d, *J* = 14.8 Hz, 1H), 7.66 (d, *J* = 7.4 Hz, 1H), 7.59 (s, 2H), 7.52 (d, *J* = 4.1 Hz, 3H), 7.44 (m, 1H), 7.41 – 7.37 (m, 1H), 7.29 (s, 2H), 7.03 – 6.95 (m, 2H), 6.45 (d, *J* = 14.9 Hz, 1H), 5.18 (s, 2H), 4.35 (q, *J* = 7.1 Hz, 2H), 2.70 (dt, *J* = 16.7, 6.0 Hz, 4H), 1.90 (q, *J* = 6.2 Hz, 2H), 1.80 (s, 6H), 1.46 (t, *J* = 7.2 Hz, 3H). ^{13}C NMR (101 MHz, Methanol-*d*$_4$) δ 177.15, 162.68, 161.70, 154.35, 145.64, 142.16, 141.10, 133.95, 133.23, 128.85, 128.63, 126.95, 126.36, 125.81, 124.09, 123.29, 122.48, 115.66, 114.19, 112.19, 111.15, 102.79, 101.14, 50.58, 40.01, 30.56, 29.34, 28.63, 26.97, 23.63, 20.23, 11.51. HRMS-ESI (m/z): $[M]^+$ calcd for $C_{34}H_{35}NO_4B^+$: 532.2654; found: 532.2660.

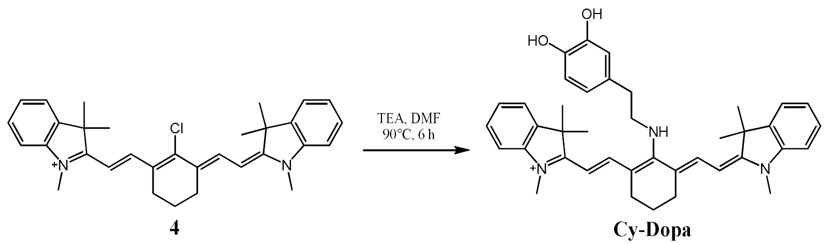

Figure S28. Synthesis of Cy-Dopa.

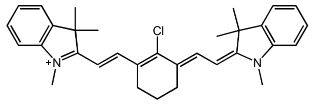

4

Compound 4 was synthesized in a similar way to compound 3. ^{1}H NMR (400 MHz, Methanol-d_4) δ 8.44 (d, *J* = 14.2 Hz, 2H), 7.52 (d, *J* = 7.4 Hz, 2H), 7.47 – 7.39 (m, 2H), 7.36 – 7.25 (m, 4H), 6.28 (d, *J* = 14.1 Hz, 2H), 3.68 (s, 6H), 2.75 (t, *J* = 6.2 Hz, 4H), 1.97 (p, *J* = 6.2 Hz, 2H), 1.74 (s, 12H). ^{13}C NMR (101 MHz, Methanol-d_4) δ 173.44, 149.51, 144.05, 142.93, 141.13, 128.46, 126.60, 125.11, 121.98, 110.65, 101.02, 49.14, 30.41, 26.80, 25.96, 20.73.

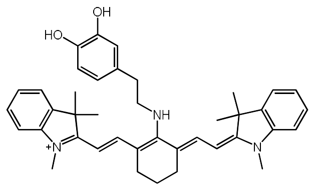

Cy-Dopa

In a dry reaction flask, compound 4 (30 mg, 0.062 mmol), dopamine hydrochloride (17.6 mg, 0.093 mmol), triethylamine (26 μL, 0.19 mmol) and anhydrous DMF (10.0 mL) as the solvent were added successively. The reaction mixture was stirred for 6 h at 90 °C. After completion of the reaction, the mixture was extracted with DCM. The combined organic layer was washed with brine (3 × 10.0 mL), and dried over anhydrous Na_2SO_4. After solvent evaporation under reduced pressure, the crude product was purified by column chromatography to provide **compound Cy-Dopa** (20 mg, 54%). ^{1}H NMR (400 MHz, Chloroform-*d*) δ 7.50 (d, *J* = 12.0 Hz, 2H), 7.38 – 7.18 (m, 7H), 7.03 (dd, *J* = 28.6, 7.1 Hz, 4H), 6.88 (d, *J* = 7.8 Hz, 2H), 6.60 (d, *J* = 7.7 Hz, 1H), 5.54 (d, *J* = 12.8 Hz, 2H), 4.02 (s, 2H), 3.39 (s, 6H), 3.00 (d, *J* = 27.2 Hz, 2H), 2.44 (s, 4H), 1.78 – 1.69 (m, 2H), 1.55 (s, 12H). ^{13}C NMR (101 MHz, Chloroform-*d*) δ 145.09, 143.97, 139.65, 137.50, 133.29, 128.16, 124.57, 123.70, 123.02, 122.28, 120.22, 115.74, 108.42, 94.60, 50.20, 47.77, 36.08, 35.00, 31.53, 30.15, 29.71, 28.85, 25.37, 21.19. HRMS-ESI (m/z): $[M]^+$ calcd for $C_{40}H_{46}N_3O_2^+$: 600.3585; found: 600.3599.

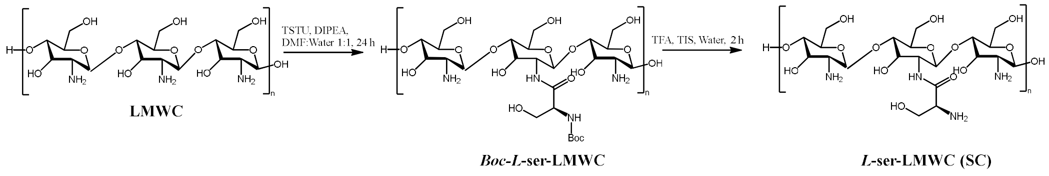

Figure S29. Synthesis of *L*-ser-LMWC (SC).

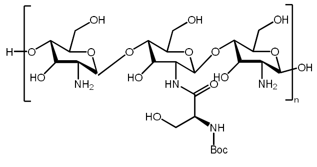

Boc-L-ser-LMWC

In a dry reaction flask, *Boc-L*-serine (102 mg, 0.5 mmol), *N*,*N*,*N*′,*N*′-TetraMethyl-*O*-(*N*-succiniMidyl)uroniuM tetrafluoroborate (TSTU) (150 mg, 0.5 mmol), DIPEA (261 μL, 1.5 mmol) and DMF (10.0 mL) as the solvent were added successively. After stirring for 4 h, the deionized water solution of LMWC (MW = 1.0 kDa, 125 mg, 0.125 mmol) was added to the mixture solution. The reaction mixture was stirred at room temperature for 24 h and then purified by dialysis [molecular

weight cutoff (MWCO), 2.0 kDa] against deionized water for 48 h, followed by lyophilization. The successful modification of *Boc*-*L*-serine can be confirmed by the characteristic peak at 1.46 ppm in the ^{1}H NMR spectrum.

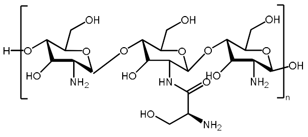

***L*-ser-LMWC (SC)**

The products were dissolved in a TFA cocktail (95% TFA, 2.5% TIS, and 2.5% water) to deprotect the *Boc* group. The reaction mixtures were stirred at room temperature for 30 min. After completion of deprotection, this solution was evaporated to remove most of TFA. The residue was purified by dialysis (MWCO, 2.0 kDa) against deionized water for 48 h, followed by lyophilization.

3. Supplementary spectra

The NMR spectra of compound **2**

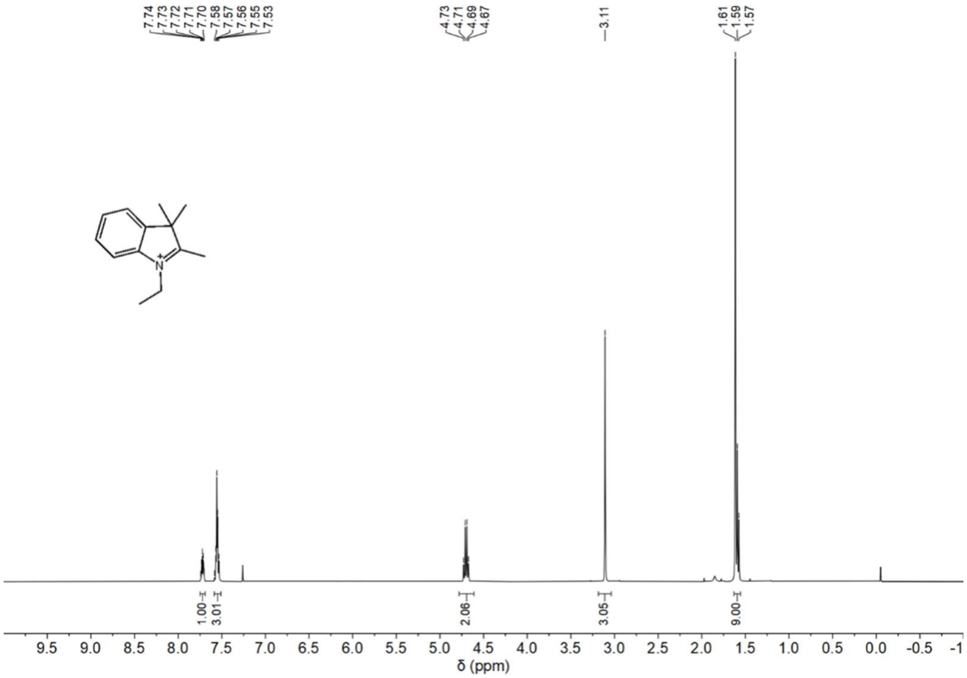

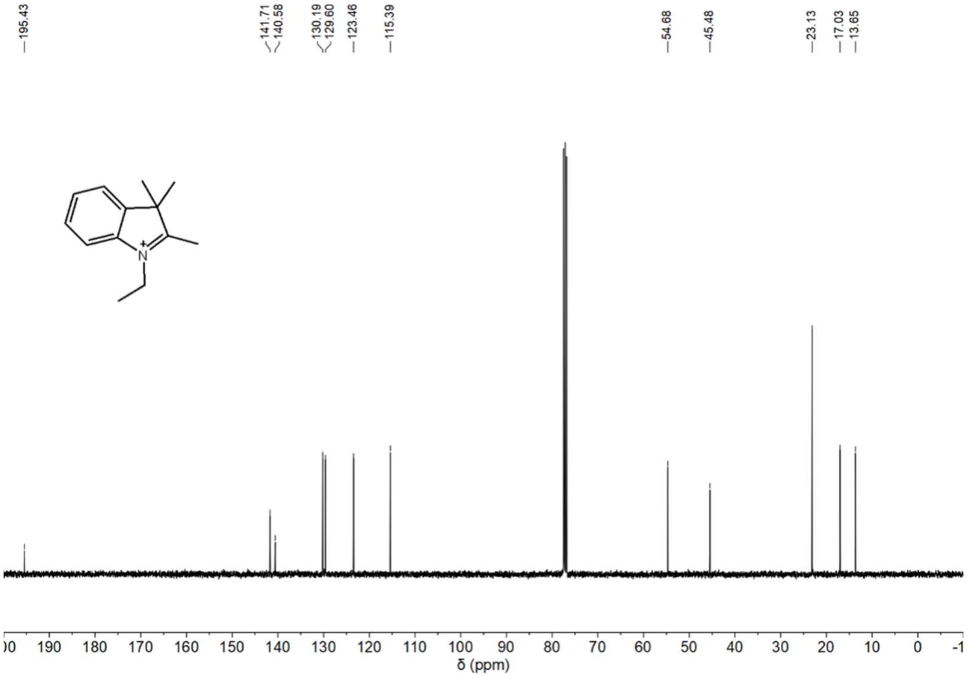

The NMR spectra of compound **3**

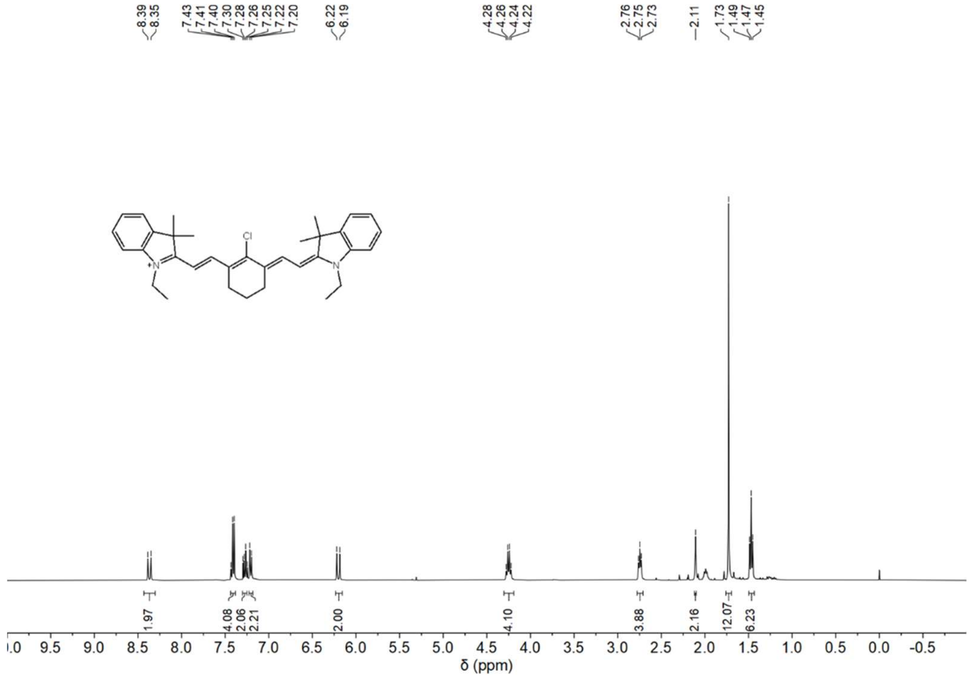

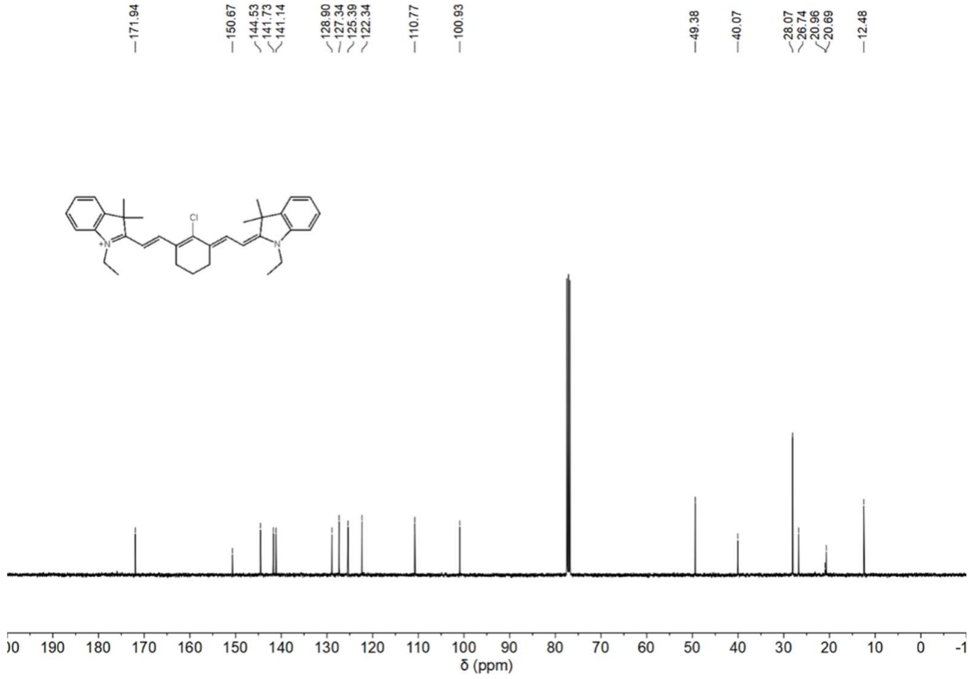

The NMR spectra of compound **Hcy-OH**

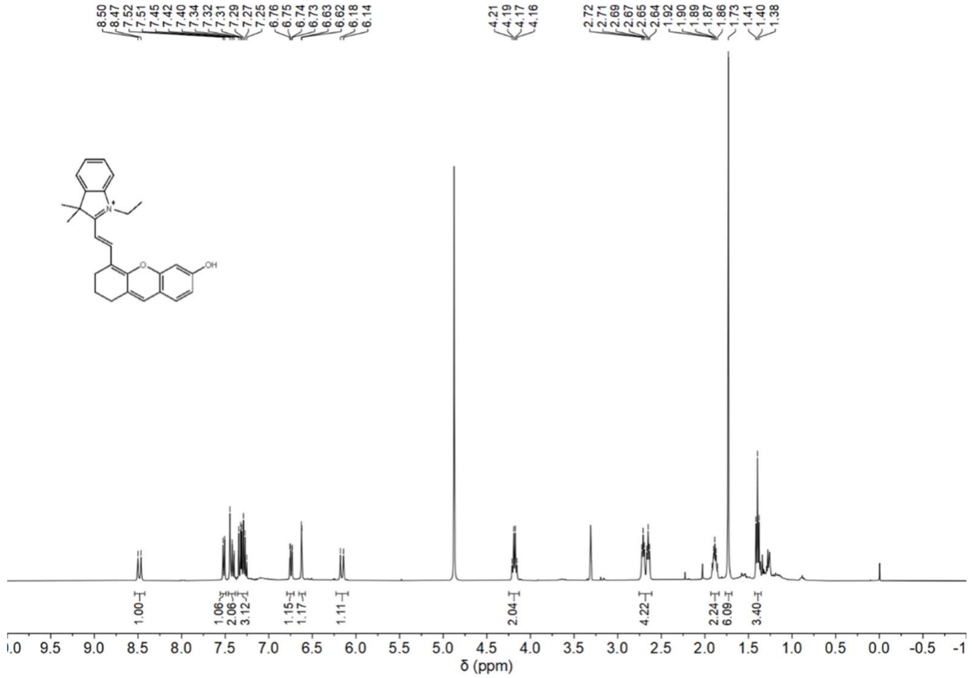

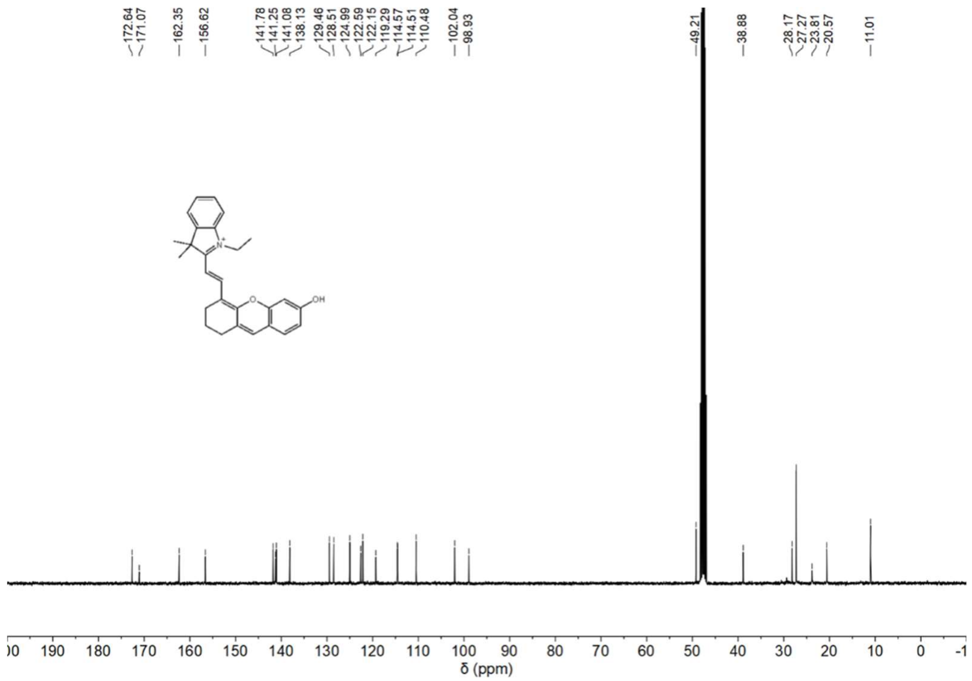

The NMR spectra of compound **Hcy-BOH**

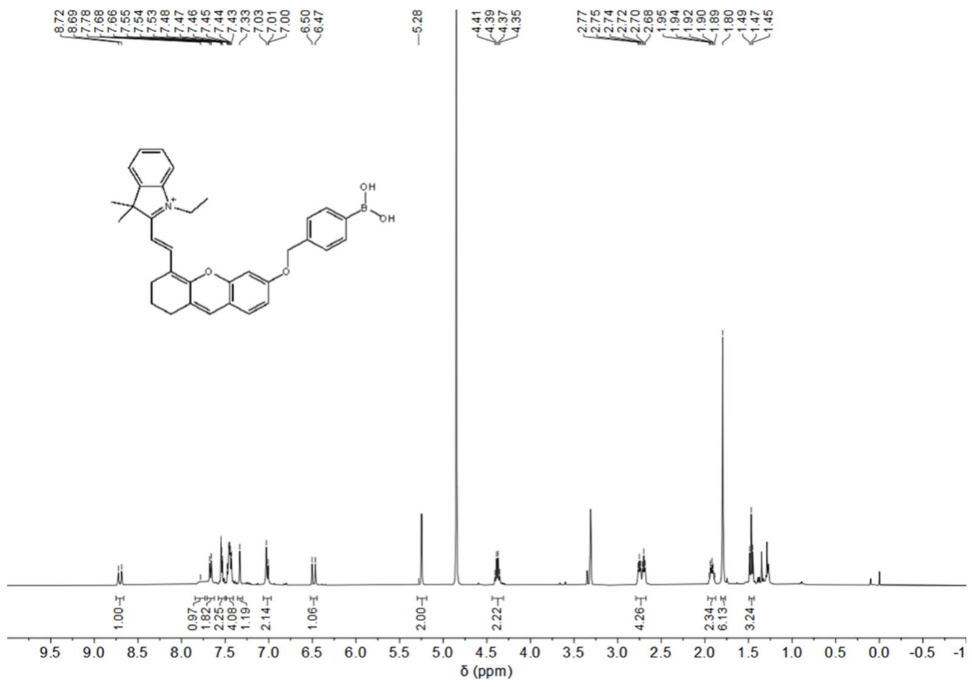

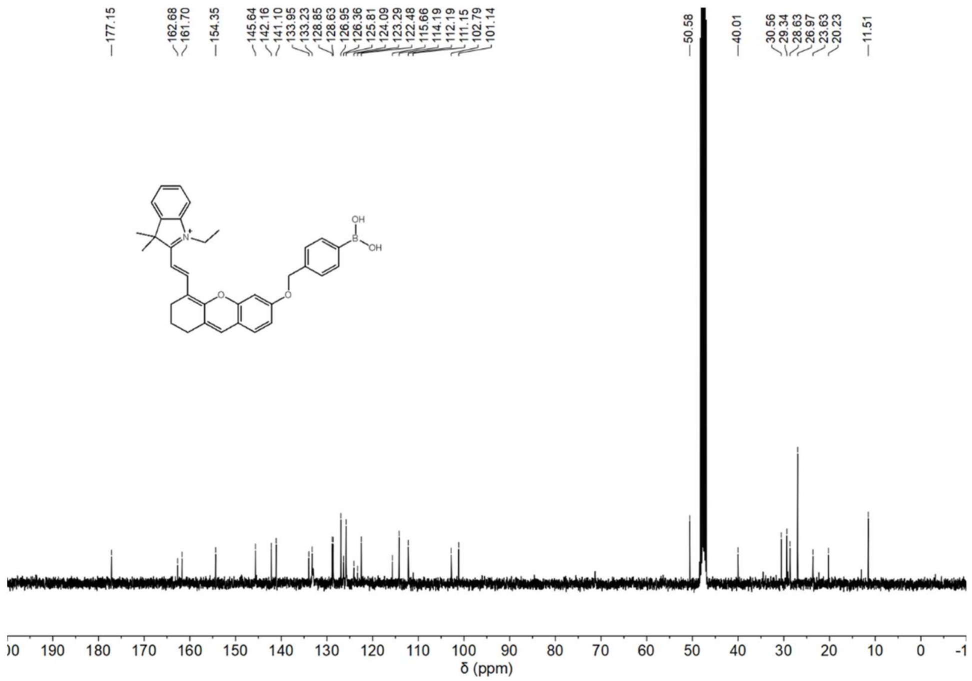

The NMR spectra of compound **4**

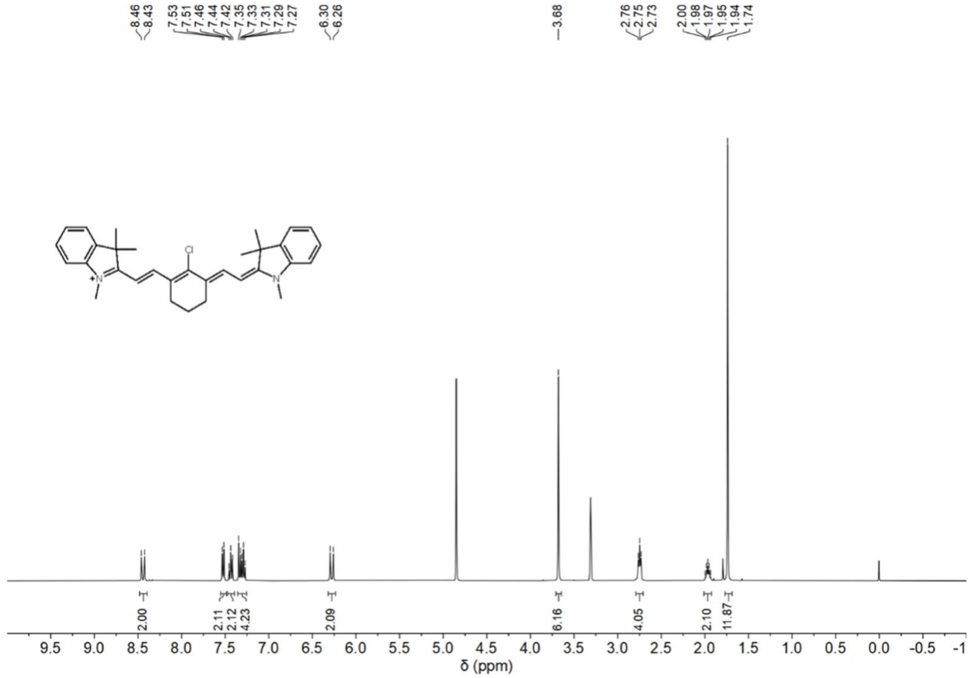

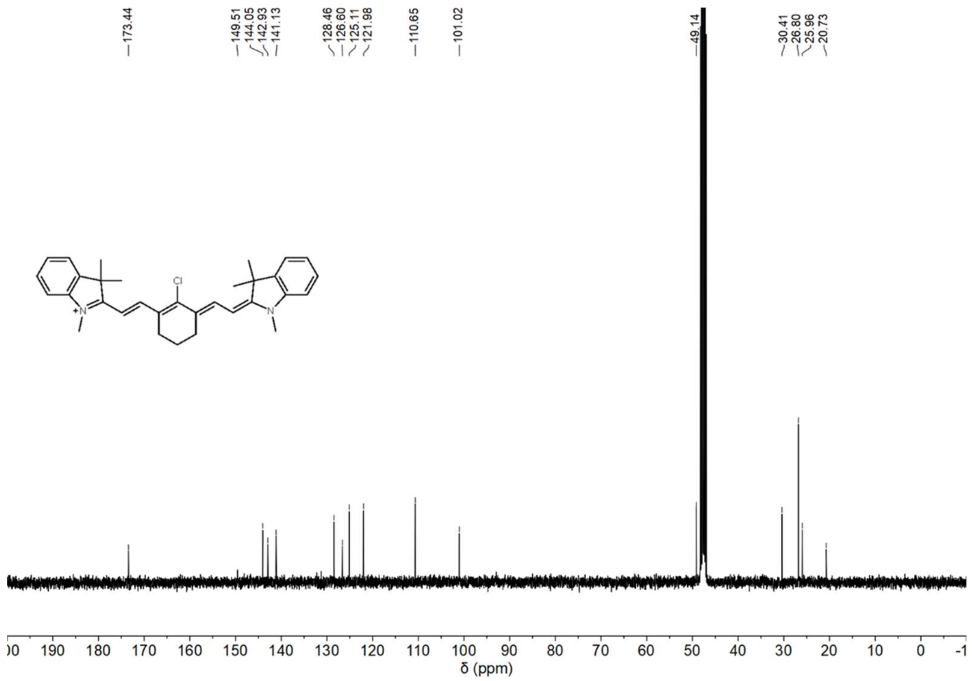

The NMR spectra of compound **Cy-Dopa**

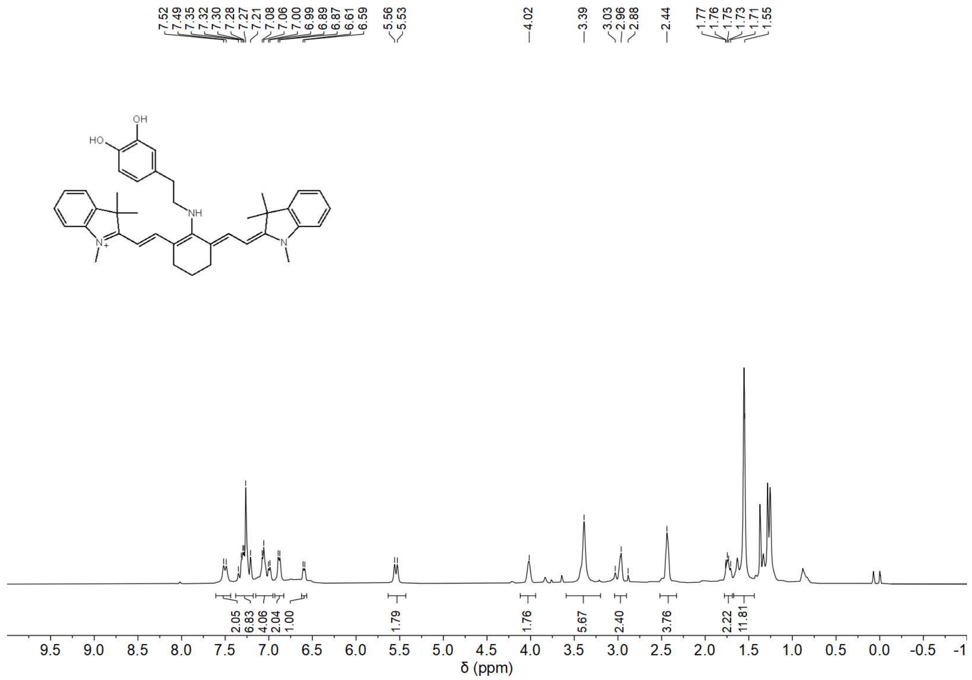

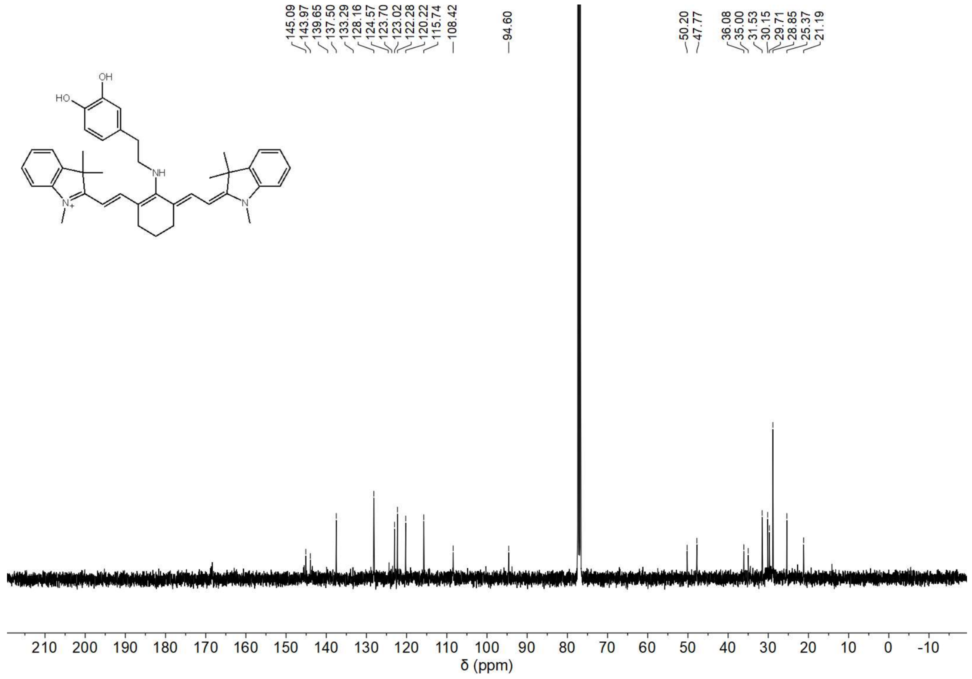

The NMR spectra of compound ***Boc-L*-serine (*Boc-L*-ser)**

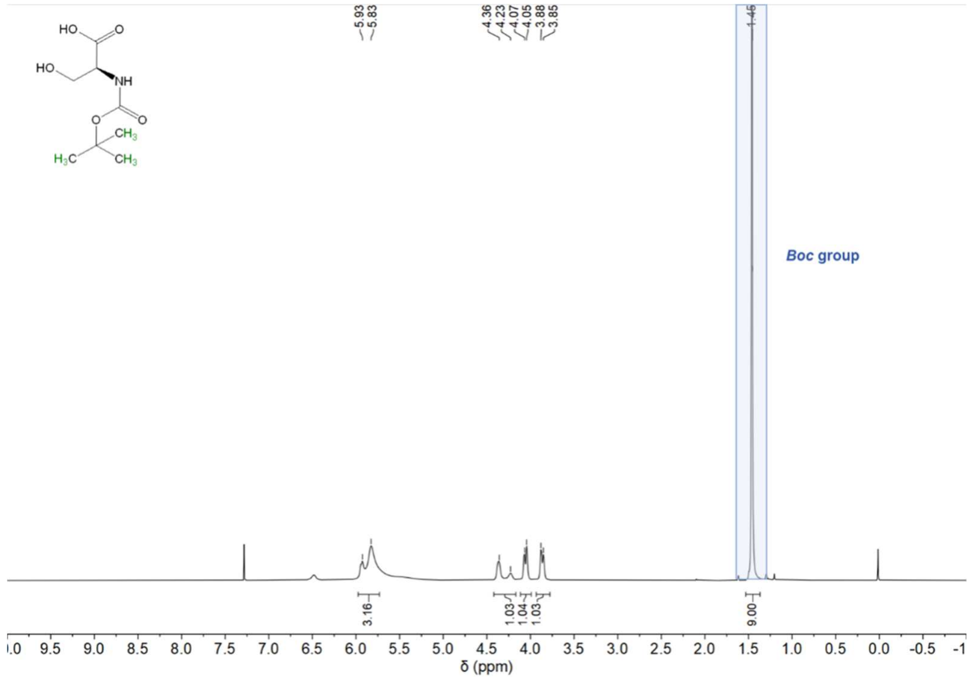

The NMR spectra of compound **LMWC**

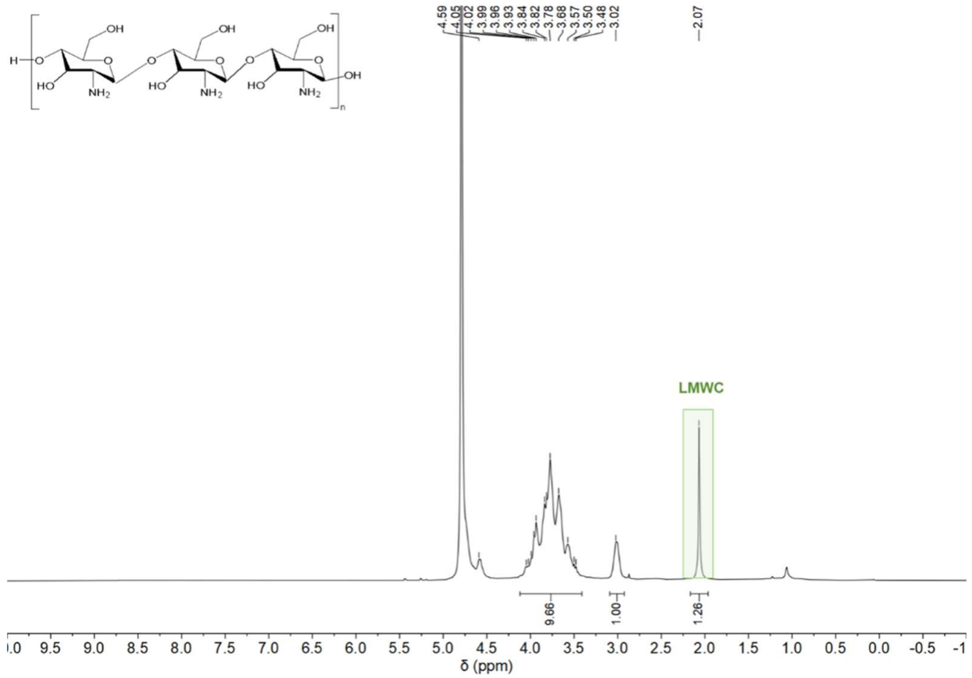

The NMR spectra of compound ***Boc-L*-serine-LMWC (*Boc-L*-ser-LMWC)**

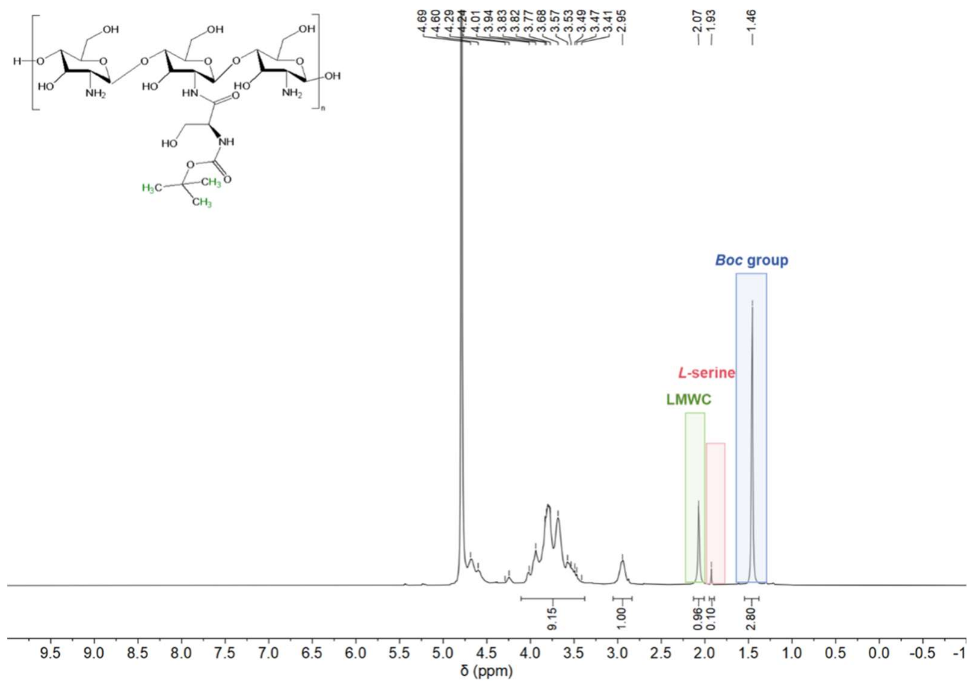

The NMR spectra of compound ***L*-serine-LMWC (*S*C)**

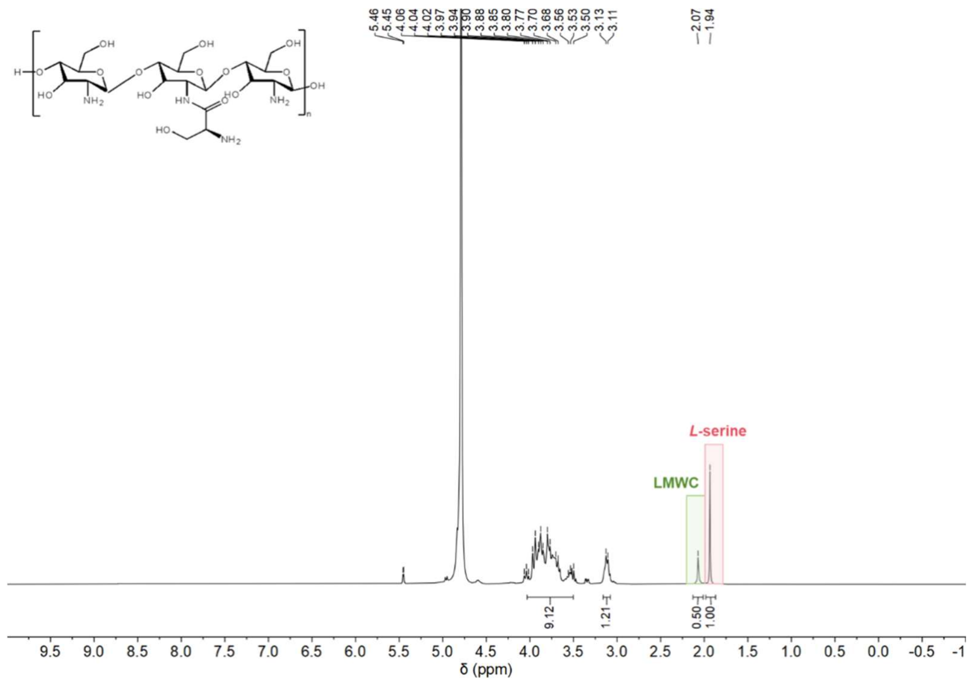

The HRMS of compound **Hcy-BOH**

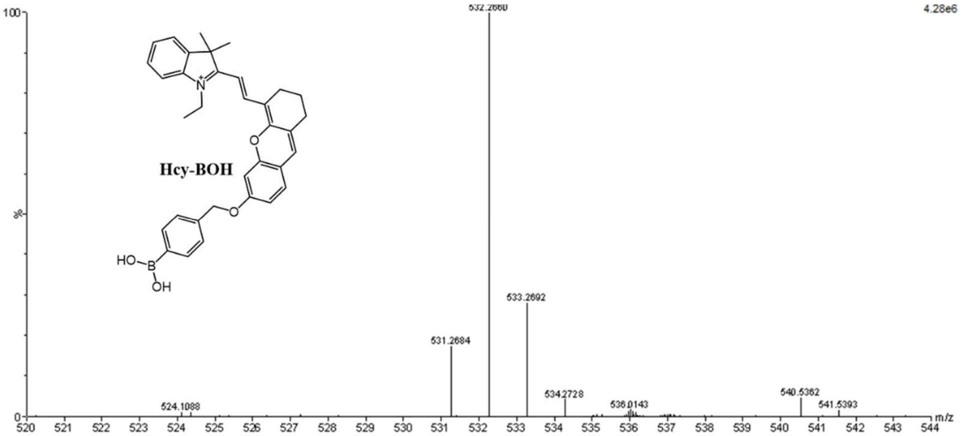

Mass	Calc. Mass	mDa	PPM	DBE	i-FIT	Norm	Conf(%)	Formula
532.2660	532.2654	0.1	0.2	18.5	547.4	n/a	n/a	$C_{34}H_{35}NO_4B$

The HRMS of compound **Cy-Dopa**

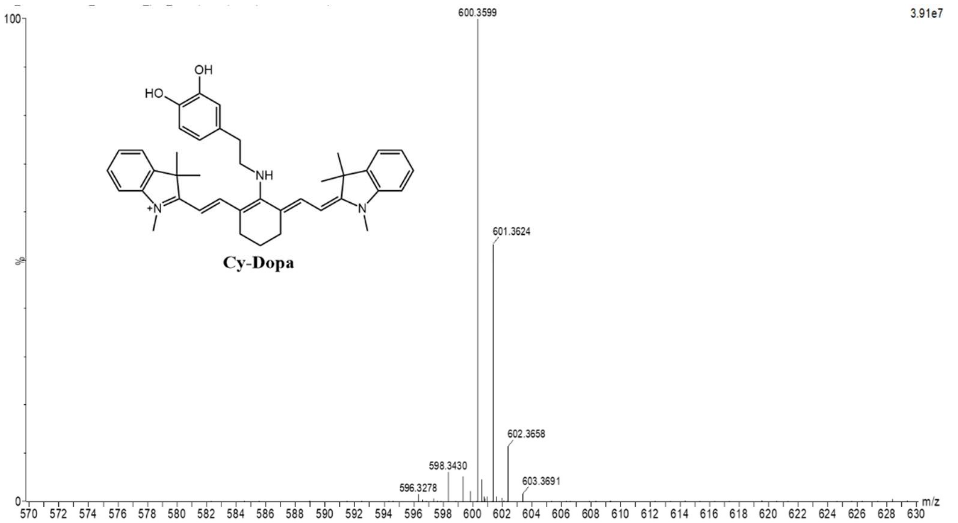

Mass	Calc. Mass	mDa	PPM	DBE	i-FIT	Norm	Conf(%)	Formula
600.3599	600.3585	0.9	1.5	19.5	762.2	n/a	n/a	$C_{40}H_{46}N_3O_2$
